# Methodological challenges, solution pathways, and future priorities in Mendelian randomization research: a bibliometric and content analysis

**DOI:** 10.3389/fgene.2026.1853737

**Published:** 2026-10-26

**Authors:** Jiapeng Zhang, Ying Xiao, Feifei Feng, Meiyi Feng, Dan Jiang, Ling Wang

**Affiliations:** 1 Faculty of Medicine, Macau University of Science and Technology, Taipa, Macau, China; 2 School of Medicine, Jiaying University, Meizhou, Guangdong, China; 3 College of Public Health, Zhengzhou University, Zhengzhou, Henan, China; 4 Emergency Department, Guangdong Provincial Hospital of Chinese Medicine, Guangzhou, Guangdong, China; 5 Department of Nephrology, People’s Hospital of Longhua, Shenzhen, Guangdong, China

**Keywords:** bibliometric analysis, causal inference, knowledge mapping, mendelian randomization, methodological challenges, research trends, solution pathways

## Abstract

**Objective:**

To characterize the global landscape, intellectual structure, major methodological challenges, dominant solution pathways, and future priorities in Mendelian randomization (MR) research.

**Methods:**

Articles and reviews on MR within the predefined 2003–2025 eligibility window were retrieved from the Web of Science Core Collection (WOSCC) and Scopus to construct a primary corpus (A-corpus). After field harmonization and hierarchical deduplication, conventional bibliometric analyses were performed. A problem-driven B-corpus was constructed by applying a prespecified terminology dictionary to titles, abstracts, and keywords, followed by manual eligibility review of dictionary-positive records and independent double coding across four dimensions: methodological challenges, solution pathways, future priorities, and solution orientations.

**Results:**

The A-corpus comprised 21,346 publications retrieved under the predefined 2003–2025 eligibility window; a small number of eligible records carried 2026 early-access or indexing metadata in the source databases. Strict dictionary screening identified 10,598 candidate records; 10,291 were excluded after eligibility review and 307 publications were retained in the final B-corpus. In the B-corpus, horizontal pleiotropy was the most frequent methodological challenge (90/307, 29.3%), mixed sensitivity framework was the dominant solution pathway (107/307, 34.9%), and reproducible reporting pipeline (45/307, 14.7%), mechanism-aware inference (36/307, 11.7%), and omics integration (34/307, 11.1%) were the leading explicitly coded future-priority categories. Inter-coder reliability ranged from moderate to almost perfect across dimensions, with Cohen’s kappa values of 0.434 for methodological challenges, 0.531 for solution pathways, 0.703 for future priorities, and 0.897 for solution orientations.

**Conclusion:**

This combined bibliometric and content analysis identified major publication patterns, recurrent methodological challenges, commonly reported solution pathways, and explicitly stated future priorities in MR research. The findings show the continuing prominence of causal-validity concerns alongside attention to mechanistic interpretation, reproducibility, generalizability, and translation; they describe patterns in the published literature rather than establishing the comparative scientific validity or clinical value of individual MR methods.

## 1 Introduction

Mendelian randomization (MR) uses genetic variants as instrumental variables to infer the causal effect of an exposure on an outcome. Because germline genetic variants are allocated before disease onset, MR can reduce confounding and reverse causation that often distort conventional observational analyses; nevertheless, its validity depends on the instrumental-variable assumptions of relevance, independence from confounding, and exclusion restriction (Davey Smith and Ebrahim, 2003; Lawlor et al., 2008; Davies et al., 2018; Davey Smith and Hemani, 2014).

The conceptual origins of MR are often traced to Katan’s proposal to use ApoE genetic variation to examine the relationship between cholesterol and cancer (Katan, 1986). Over the past 2 decades, larger genome-wide association studies (GWAS), biobank resources, open summary statistics, and methodological developments have enabled MR to be applied across a wider range of exposures, outcomes, and study designs (Burgess et al., 2015; Hemani et al., 2018). These applications include drug-target MR, multivariable MR (MVMR), mediation MR, nonlinear MR, summary-data-based molecular prioritization, colocalization, within-family designs, reporting guidelines, multi-omic MR, and genetic drug-target validation (Gill et al., 2021; Sanderson et al., 2019; Carter et al., 2021; Staley and Burgess, 2017; Zhu et al., 2016; Giambartolomei et al., 2014; Brumpton et al., 2020; Skrivankova et al., 2021a; Cupi et al., 2024; Schmidt et al., 2020). These approaches address distinct inferential questions and potential sources of bias; their increasing presence in the published literature should not by itself be interpreted as evidence that individual causal estimates, or the field as a whole, have become methodologically more valid.

The expanding range of MR applications creates a need to distinguish publication growth from evidence about methodological validity and to examine how the literature frames recurring challenges, proposed solutions, and future priorities. Bibliometric and knowledge-mapping approaches use co-occurrence analysis, co-citation analysis, clustering, and burst detection to describe knowledge structures, collaboration patterns, and changes in the prominence of published topics. Software platforms such as CiteSpace, VOSviewer, and bibliometrix provide complementary tools for visualizing bibliometric networks and science-mapping results, while PRISMA-style flow reporting can make screening and exclusion procedures more transparent (Chen, 2006; van Eck and Waltman, 2010; Aria and Cuccurullo, 2017; Page et al., 2021). These approaches describe the published literature but do not evaluate the validity of individual MR findings.

Most existing reviews in MR have focused on specific diseases, particular methods, or single application scenarios. Recent reviews and bibliometric studies have described publication growth and applications in areas such as cardiovascular disease and psychiatry, while updated guidance has emphasized transparent design, instrument selection, sensitivity analysis, and reporting (Zhong and Cheng, 2024; Saccaro et al., 2022; Burgess et al., 2023). Compared with studies centered mainly on publication volume, disease-specific applications, or thematic hotspots, an integrated analysis linking science mapping to a prespecified, problem-oriented synthesis of methodological challenges, proposed solutions, and explicitly stated future priorities remains limited.

Accordingly, this study used two linked corpora with distinct purposes. The A-corpus preserved breadth and was used to map publication output, collaboration patterns, the intellectual base, and thematic evolution across the eligible MR literature. The B-corpus preserved interpretive depth and was restricted to publications that explicitly addressed methodological challenges, solution strategies, reporting or reproducibility issues, or future priorities. The study addressed four corresponding questions: (1) Which methodological challenges were most frequently represented? (2) Which solution pathways were most often reported? (3) Which future priorities were explicitly stated? and (4) How were publications distributed across validity-first, mechanism-aware, generalizability-enhanced, translation-ready, and hybrid solution orientations? This two-tier design links macro-level bibliometric mapping with manually adjudicated, problem-oriented content analysis.

## 2 Methods

### 2.1 Data sources and search strategy

This study used the Web of Science Core Collection (WOSCC) and Scopus as source databases. To maximize topical specificity, the search strategy was restricted to the exact core terms “Mendelian randomization” and “Mendelian randomisation” and did not include broader terms related to causal inference or genetic epidemiology. The eligibility window covered publications from 1 January 2003, to 31 December 2025, and was limited to English-language articles and reviews. Both databases were searched on 15 February 2026. A small number of eligible records carried 2026 early-access or indexing metadata in the exported database fields; these metadata were retained for traceability but did not redefine the prespecified 2003–2025 eligibility window.

The WOSCC search query was as follows:

TS=(“Mendelian randomization” OR “Mendelian randomisation” OR “Mendelian randomization analysis” OR “Mendelian randomisation analysis”) AND LA=(English) AND DT=(Article OR Review).

The Scopus search query was as follows:

TITLE-ABS-KEY (“Mendelian randomization” OR “Mendelian randomisation” OR “Mendelian randomization analysis” OR “Mendelian randomisation analysis”) AND (LIMIT-TO (DOCTYPE, “ar”) OR LIMIT-TO (DOCTYPE, “re”)) AND LIMIT-TO (LANGUAGE, “English”) AND PUBYEAR >2002 AND PUBYEAR <2026.

WOSCC records were exported in plain-text format and Scopus records in BibTeX format, with full bibliographic information retained for subsequent integration, deduplication, and visualization.

### 2.2 Data processing and deduplication

After retrieval, records from WOSCC and Scopus were imported into R for unified processing. Preprocessing included field harmonization, source tagging, text normalization, and hierarchical deduplication. Original database identifiers and source-file information were retained throughout the workflow to preserve source traceability.

Deduplication was performed hierarchically. First, exact duplicates were removed using DOI as the primary identifier after standardizing the DOI field by converting it to lowercase, removing URL prefixes, and deleting the leading label “doi”. Records that could not be matched by DOI were then screened using standardized title–year combinations, followed by title–first author–year matching to identify residual duplicates caused by database-specific metadata differences. Titles were normalized by standardizing case, punctuation, spacing, and special characters. When multiple records were judged to represent the same publication, the version with more complete bibliographic information was retained. Records that could not be resolved confidently by rule-based matching were kept rather than removed.

The final deduplicated dataset constituted the A-corpus. This corpus was used for conventional bibliometric analyses and also served as the basis for constructing the problem-driven subcorpus (B-corpus). Data processing, integration, and preliminary analyses were conducted primarily in R, whereas knowledge maps were generated using VOSviewer, CiteSpace, and related statistical visualization tools.

### 2.3 Conventional bibliometric analysis of the A-corpus

The A-corpus was used to characterize the overall publication landscape, collaboration structure, intellectual base, and thematic evolution of MR research. Annual publication output and whole-count record frequencies for countries/regions, institutions, authors, and journals were summarized to describe participation patterns. Collaboration networks were then constructed at the country, institutional, and author levels to delineate international connections, institutional links, and author clusters.

Productivity statistics for countries/regions, institutions, authors, and journals were summarized using whole counting. Under this approach, each publication contributed one count to every participating country/region, institution, author, or journal unit identified in the bibliographic metadata. Country- and institution-level values therefore represent participation records and are not mutually exclusive fractional publication shares. Whole-count frequencies and network centrality were used only to describe participation and collaboration patterns; they were not treated as evidence that a country, institution, or author exercised methodological leadership or produced scientifically superior work.

To identify major themes and their evolution, keyword co-occurrence analysis, keyword clustering, timeline analysis, and burst detection were performed. Co-occurrence and clustering were used to identify high-frequency themes and their interrelationships; timeline mapping was used to trace the persistence and transition of major thematic clusters over time; and burst analysis was used to detect research topics that rose rapidly during specific periods.

In addition, reference co-citation analysis was performed to reveal the intellectual base and representative literature structure of MR research. This included co-citation clustering, timeline analysis, and burst detection of influential references, thereby providing a macro-level view of the field’s developmental trajectory and knowledge evolution.

### 2.4 Construction of the B-corpus

To construct a problem-driven subcorpus that could capture methodological challenges, solution pathways, and future priorities in Mendelian randomization (MR) research, we derived the B-corpus from the deduplicated A-corpus through a structured dictionary-based screening and eligibility-review procedure. The A-corpus was used to preserve the breadth of the MR literature, whereas the B-corpus was designed to provide a focused, interpretable content-analytic sample of publications that explicitly addressed methodological problems, evaluated or proposed solution pathways, discussed reporting or reproducibility standards, or articulated emerging methodological directions.

First, a terminology dictionary was developed before screening and organized into three domains: methodological challenges, solution pathways, and future priorities. Candidate terminology was drawn from primary MR methods papers, reporting guidelines, and iterative review of the methodological literature. Canonical sources covered MR-Egger, weighted median and mode-based estimators, MR-PRESSO, weak-instrument bias, sample overlap, winner’s curse, Steiger directionality, CAUSE, colocalization, within-family MR, nonlinear MR, drug-target MR, reporting standards, reproducible pipelines, and broader best-practice guidance (Burgess et al., 2023; Bowden et al., 2015; Bowden et al., 2016; Hartwig et al., 2017; Verbanck et al., 2018; Burgess et al., 2011; Burgess et al., 2016; Jiang et al., 2023; Hemani et al., 2017; Morrison et al., 2020; Wallace, 2021; Davies et al., 2019; Haycock et al., 2016; Skrivankova et al., 2021b). For each final category, the coding materials paired the included terms with an operational definition, inclusion and exclusion rules, and examples. Terms were used to flag candidate records rather than to determine final inclusion automatically; potentially ambiguous matches were resolved during manual eligibility review. The complete dictionary and category-level decision rules are provided in Supplementary Table S1.

Second, the dictionary was applied to the titles, abstracts, and keywords of all records in the A-corpus. Of the 21,346 deduplicated A-corpus records, 10,598 matched at least one strict dictionary term and were retained as candidates for manual eligibility review. A dictionary match was therefore a screening flag, not proof of eligibility. Records without a strict match were not carried forward because the available title, abstract, and keyword fields did not contain prespecified evidence of a primary focus on an MR methodological challenge, solution pathway, or future priority.

Third, candidate records were evaluated using title, abstract, keyword, and, where necessary, full-text information. Publications were included in the B-corpus if they met at least one of the following criteria: (1) the primary focus was an MR methodological challenge, such as horizontal pleiotropy, weak instruments, sample overlap, population stratification, reverse causation, family-related bias, or molecular attribution; (2) the publication evaluated, compared, developed, or systematically discussed a methodological solution pathway, such as pleiotropy-robust estimators, multivariable MR, colocalization, within-family designs, nonlinear MR, or reporting guidelines; (3) the article focused on reproducibility, reporting transparency, or methodological standardization in MR; or (4) the article explicitly discussed future methodological priorities, such as multi-ancestry validation, multi-omics integration, mechanism-aware inference, triangulation with external evidence, or clinical and public-health translation.”

Records were excluded if they were routine exposure-outcome MR applications that only reported standard sensitivity analyses without a primary methodological focus; omics or drug-target MR applications without methodological evaluation; GWAS or resource studies in which MR was only a secondary or peripheral component; topic-specific reviews that summarized disease associations rather than MR methodology; non-eligible publication types such as protocols or commentaries without substantive methodological synthesis; or records with no explicit MR methodological challenge, solution pathway, or future-priority focus. Of the 10,598 dictionary-positive candidates, 10,291 were excluded and 307 were retained. Supplementary Table S2 and the accompanying screening workbook report the stage-specific screening counts, hierarchical primary exclusion categories, operational rationales, and reconciliation totals. These materials provide aggregate auditability of the A-corpus-to-B-corpus workflow and are not presented as a record-level decision log.

After eligibility review, 307 publications were retained as the final B-corpus and were used for problem-driven content coding. The screening process is summarized in Figure 1 and Supplementary Table S2.

**FIGURE 1:**
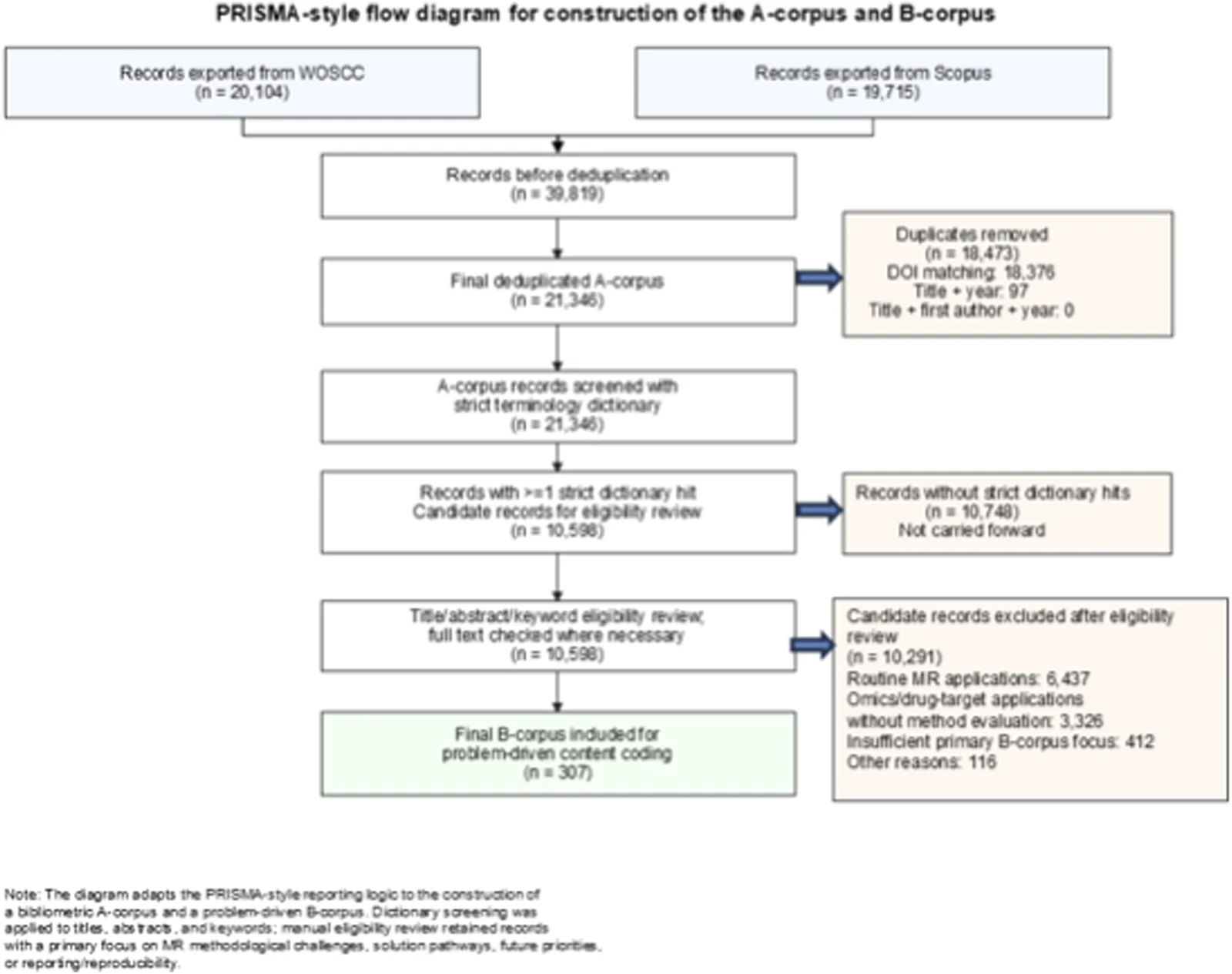
PRISMA-style flow diagram for construction of the A-corpus and B-corpus. The diagram summarizes database retrieval, deduplication, strict dictionary-based screening, manual eligibility review, exclusion categories, and the final B-corpus retained for problem-driven content coding.

### 2.5 Problem-driven content coding and reliability assessment

To enhance the interpretability and reproducibility of the B-corpus analysis, we developed a prespecified problem-driven content-coding framework and classified each included publication across four dimensions: methodological challenges, solution pathways, future priorities, and solution orientations. Each publication was treated as one coding unit, and one primary code was assigned within each dimension according to its dominant methodological emphasis; secondary considerations were retained in the coding notes. This rule produced mutually interpretable frequency distributions and cross-tabulations and avoided inflating counts when one publication addressed several related topics. It was not intended to capture the full multiplicity of each publication, and secondary challenges, solutions, or priorities may consequently be underrepresented. The “hybrid” orientation was assigned only when two or more orientations were comparably emphasized and no single dominant orientation could be identified. “None explicitly stated” was assigned only when the analyzed text contained no future-priority statement meeting the coding criteria; it does not mean that no unresolved issue or future research need existed.

All 307 B-corpus publications were independently coded by two reviewers according to the prespecified coding manual. The second reviewer was blinded to the first reviewer’s coding decisions during independent coding. The four nominal coding dimensions were methodological challenge, solution pathway, future priority, and solution orientation. Before consensus adjudication, inter-coder reliability was assessed separately for each dimension using unweighted Cohen’s kappa, and percent agreement was reported as an additional descriptive measure.

After reliability assessment, all coding disagreements were reviewed through consensus discussion between the two coders. Disagreements were resolved by rechecking the title, abstract, keywords, key-evidence excerpt, full text where necessary, and the operational definitions in the coding manual. The final consensus code was selected according to the one-primary-code rule and the dominant methodological emphasis of the publication. The final frequency analyses, cross-tabulations, and synthesis framework were based on the consensus coding table after adjudication.

### 2.6 Statistical analysis and visualization

Data integration, deduplication, and part of the statistical aggregation were conducted primarily in R (version 4.5.2). VOSviewer (version 1.6.20) was used mainly to construct and visualize country-, institution-, and author-level collaboration networks as well as keyword co-occurrence networks. CiteSpace (version 6.4) was used mainly for keyword clustering, timeline analysis, burst detection, and reference co-citation analysis, whereas R and bibliometrix-related workflows were further used for frequency statistics, cross-tabulations, and graphical presentation of the coded B-corpus results (Chen, 2006; van Eck and Waltman, 2010; Aria and Cuccurullo, 2017).

To improve reproducibility, the main parameter settings for each bibliometric and visualization analysis are reported in Supplementary Table S4. For VOSviewer analyses, we recorded the unit of analysis, counting method, minimum document or occurrence threshold, normalization method, clustering resolution, and network metrics. For CiteSpace analyses, we recorded the time span, slice length, node type, selection criterion, pruning strategy, burst-detection settings, and network metrics where available. Cited-reference variants were manually inspected and merged using the CiteSpace alias function before regenerating the reference co-citation network.

Within the problem-driven analysis of the B-corpus, frequencies and proportions were calculated for the consensus primary codes across the four dimensions. Cross-tabulations were then used to summarize the relationships between methodological challenges and solution pathways, and these patterns were visualized as heatmaps. Temporal distributions of future-priority codes were summarized by publication year. Finally, the four-dimensional coding results were synthesized into a challenge-solution-gap-priority matrix and a descriptive solution-orientation framework. Reader-facing labels in Figures 2–4 use the standard category names defined in Supplementary Table S1; internal underscore-style codes were converted only for display.

**FIGURE 2:**
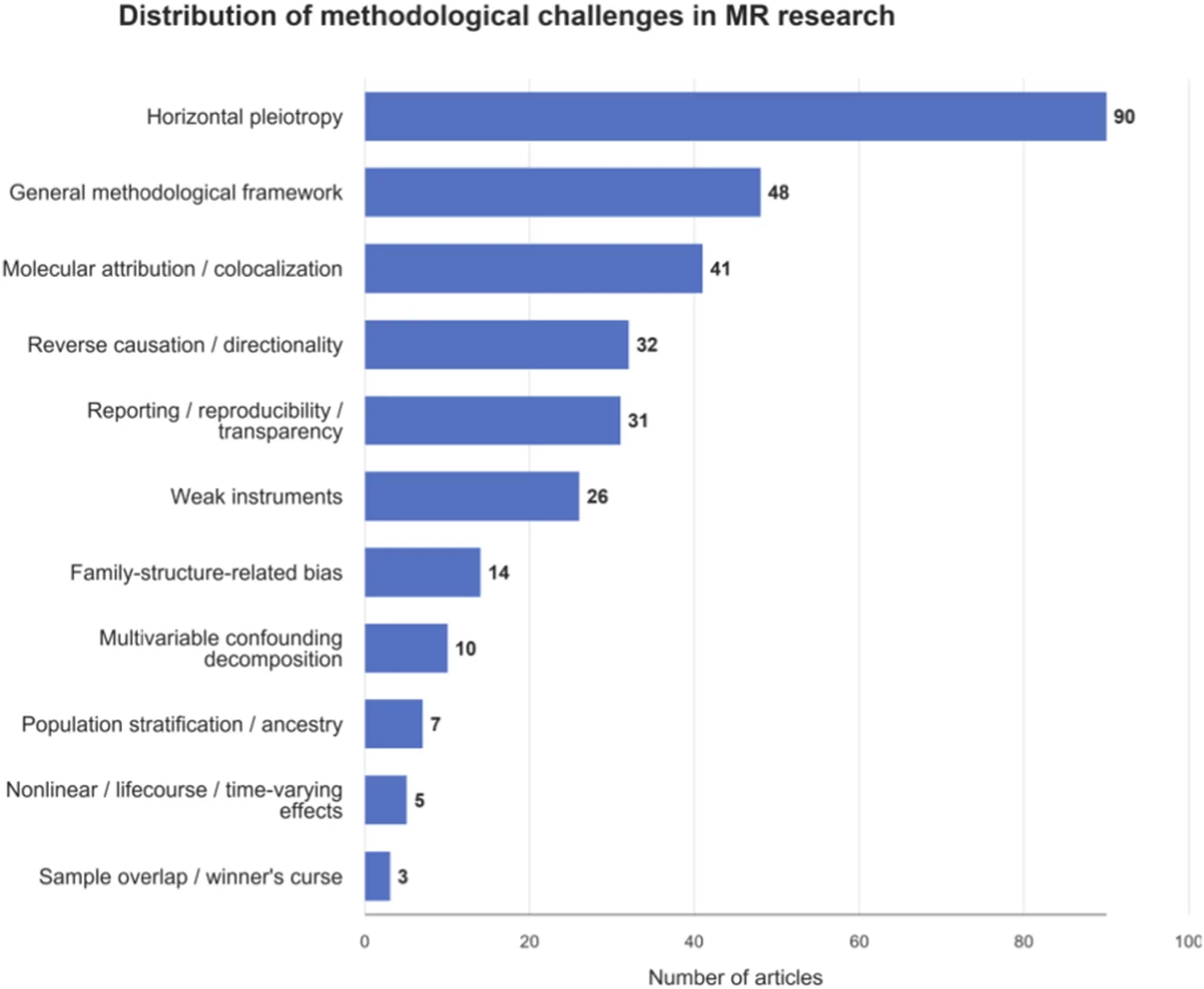
Distribution of primary methodological-challenge codes in the B-corpus (n = 307). Each publication contributes one primary code according to its dominant methodological focus; secondary issues may therefore be underrepresented.

**FIGURE 3:**
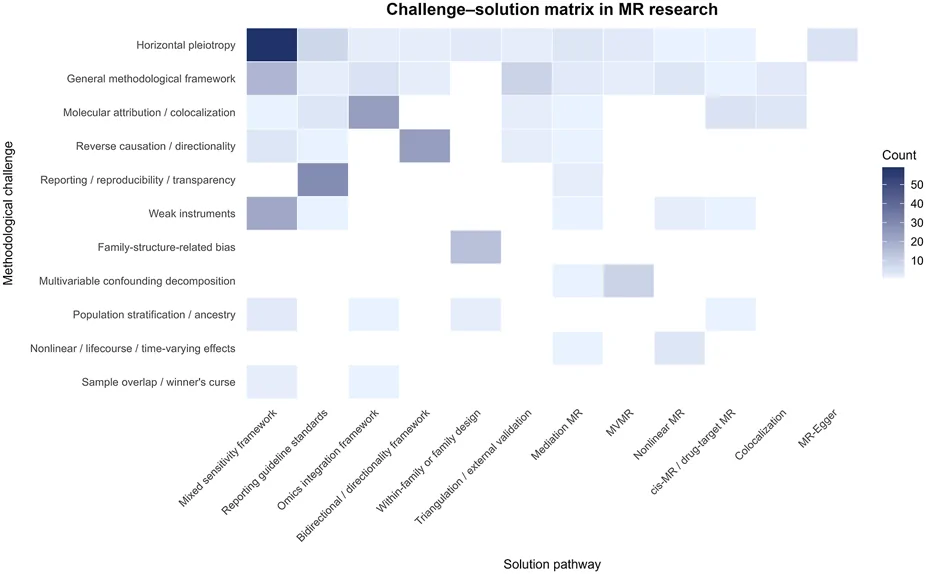
Cross-tabulation heatmap of primary methodological-challenge and solution-pathway codes in the B-corpus (n = 307). Cell color intensity represents the number of publications assigned to each challenge–solution pairing. Blank cells indicate zero publications. Each publication contributes one primary code per dimension.

**FIGURE 4:**
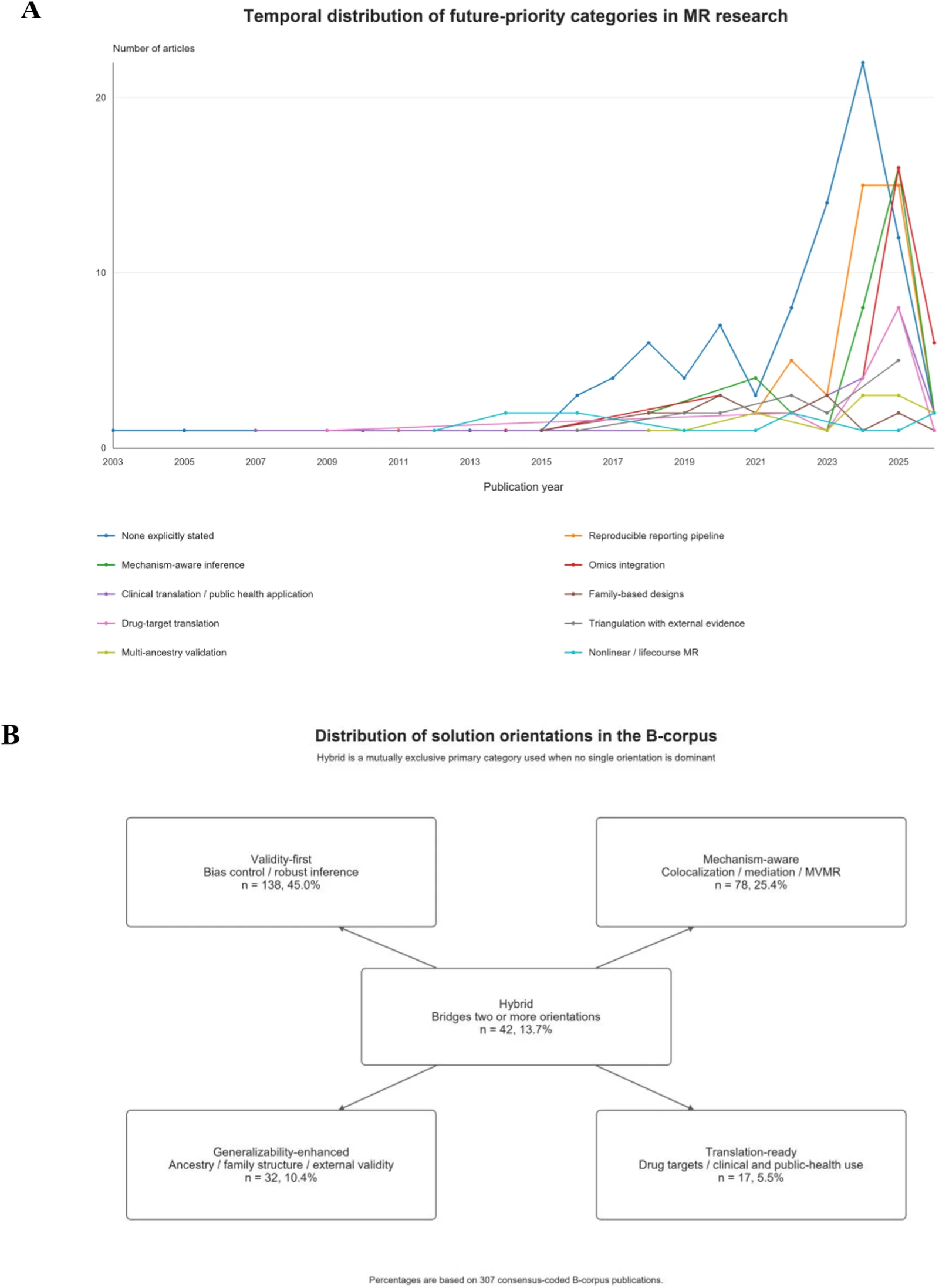
Future-priority codes and descriptive solution-orientation categories in the B-corpus (n = 307). **(A)** Annual distribution of the ten future-priority categories defined in Supplementary Table S1. “None explicitly stated” denotes absence of an explicit codable statement and does not imply absence of future research needs. Lines connect observed annual category counts and are descriptive rather than evidence of a developmental transition. **(B)** Frequencies of the validity-first, mechanism-aware, generalizability-enhanced, translation-ready, and hybrid primary orientation codes; hybrid is a mutually exclusive category assigned when two or more orientations were comparably emphasized and no single orientation was dominant.

## 3 Results

### 3.1 Dataset overview and annual growth

According to the predefined search strategy (Table 1), MR-related publications were retrieved from WOSCC and Scopus for the 2003–2025 eligibility window. After field harmonization, merging, and hierarchical deduplication, the final A-corpus comprised 21,346 publications. A small number of eligible records carried 2026 early-access or indexing metadata in the source databases; the eligibility period itself remained 2003–2025. The corpus involved 2,407 journals, 53,126 authors, 109 countries/regions, and 3,828 institutions (Table 2).

**TABLE 1:** Search strategy and data sources for the A-Corpus.

Database	Publication time span	Language	Document types	Retrieval date	Search query
Web of science core collection (WOSCC)	1 January 2003–31 December 2025	English	Article; review	15 February 2026	TS=(“Mendelian randomization” OR “mendelian randomisation” OR “mendelian randomization analysis” OR “mendelian randomisation analysis”) AND LA=(English) AND DT=(Article OR review)
Scopus	1 January 2003–31 December 2025	English	Article; review	15 February 2026	TITLE-ABS-KEY (“Mendelian randomization” OR “mendelian randomisation” OR “mendelian randomization analysis” OR “mendelian randomisation analysis”) AND (LIMIT-TO (DOCTYPE, “ar”) OR LIMIT-TO (DOCTYPE, “re”)) AND LIMIT-TO (LANGUAGE, “English”) AND PUBYEAR >2002 AND PUBYEAR <2026

The prespecified publication eligibility window was 2003–2025. A small number of eligible records carried 2026 early-access or indexing metadata in the source databases; those metadata did not redefine the eligibility window.

**TABLE 2:** Main characteristics of the A-Corpus after deduplication.

Characteristic	Value
Total number of records	21,346
Publication year range	2003–2025
Number of journals	2,407
Number of authors	53,126
Number of countries/regions	109
Number of institutions	3,828

The number of institutions was calculated from the standardized institutional field (AU_UN), where available. The number of countries/regions was derived from affiliation addresses (C1) after basic normalization of country labels.

On the basis of the A-corpus, a problem-driven subcorpus (B-corpus) was constructed through strict dictionary screening and manual eligibility review. The dictionary screen identified 10,598 candidates from the 21,346-record A-corpus; 10,291 candidates were excluded during eligibility review and 307 publications were retained (Figure 1; Supplementary Table S2). The A-corpus was used for the bibliometric analyses, whereas the B-corpus was used for the problem-driven content analyses.

All 307 B-corpus publications were double-coded before consensus adjudication. Inter-coder agreement was moderate for methodological challenges (percent agreement, 50.5%; Cohen’s kappa, 0.434) and solution pathways (percent agreement, 63.5%; Cohen’s kappa, 0.531), substantial for future priorities (percent agreement, 73.6%; Cohen’s kappa, 0.703), and almost perfect for solution orientations (percent agreement, 92.8%; Cohen’s kappa, 0.897). All disagreements were reviewed against the source text and operational definitions and resolved by consensus before the final frequency, cross-tabulation, and synthesis-matrix analyses (Supplementary Table S3).

Annual publication output was relatively low between 2003 and 2011, increased after 2012, accelerated after 2018, and rose further after 2020 (Figure 5). The 2025 count was lower than the 2024 count. Because retrieval occurred on 15 February 2026, the 2025 value may be affected by database indexing completeness and should be interpreted cautiously.

**FIGURE 5:**
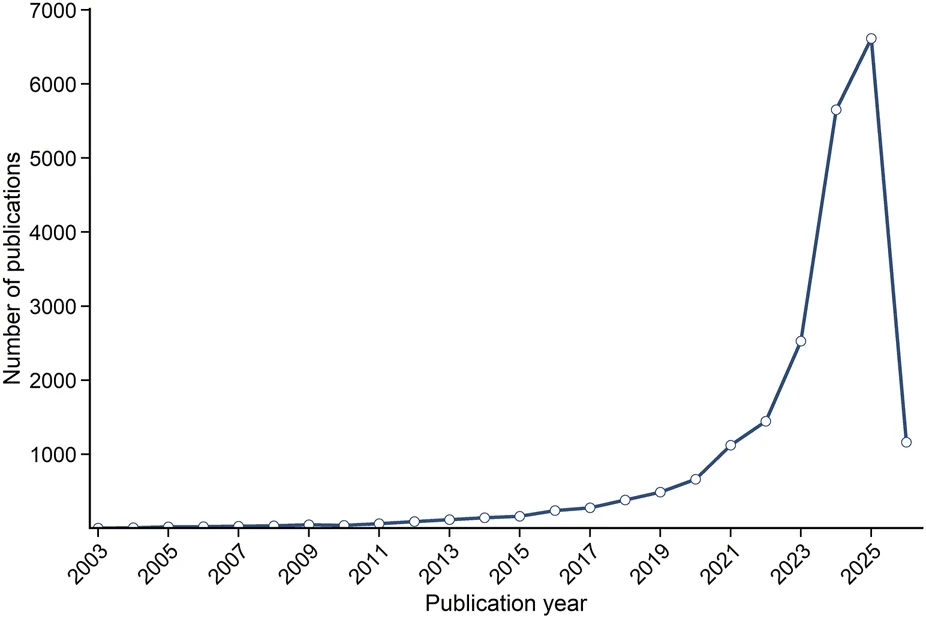
Annual publication output in the A-corpus. Counts are based on 21,346 deduplicated records retrieved under the predefined 2003–2025 eligibility window. Because retrieval occurred on 15 February 2026, the 2025 count may be affected by indexing completeness.

### 3.2 Publication participation and collaboration structure

Country- and institution-level collaboration networks showed a concentrated pattern of participation and international connection (Figure 6). Using whole counting, China had the largest number of country-level participation records (14,695), followed by the United States (3,364) and the United Kingdom (3,043), with Germany, Sweden, and the Netherlands ranking next (Table 3). Because a multi-country publication contributed one count to every participating country/region, these values are participation frequencies rather than mutually exclusive publication shares or evidence of methodological leadership. China, the United States, and the United Kingdom also had central positions in the collaboration network, reflecting participation volume and network connectedness.

**FIGURE 6:**
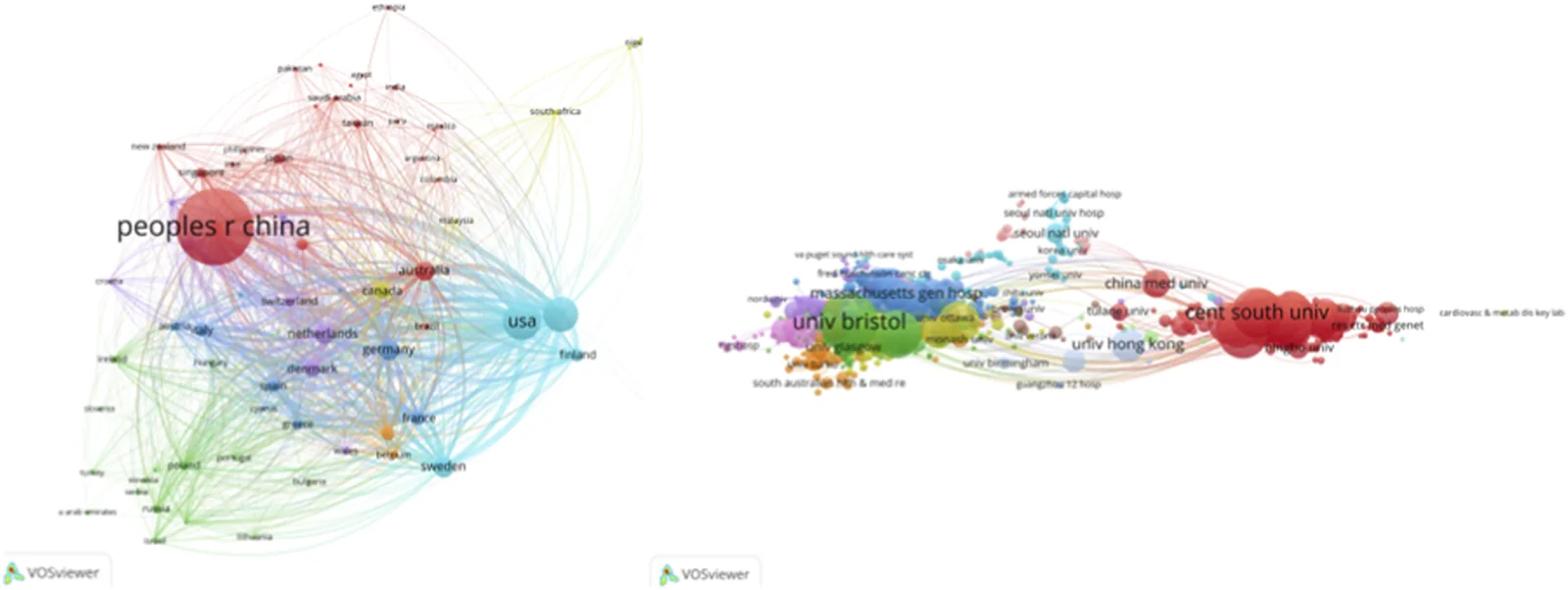
Country- and institution-level collaboration networks in the A-corpus. Networks were generated using whole counting; node prominence reflects record frequency and network connections reflect bibliographic collaboration. Whole-count values describe participation and connectedness, not fractional publication shares or methodological leadership.

**TABLE 3:** Countries/regions, institutions, authors, and journals with the highest whole-count record frequencies.

Panel A. Top countries/regions			Panel B. Top institutions			Panel C. Top authors			Panel D. Top journals		
Rank	Country/region	Whole-count records	Rank	Institution	Whole-count records	Rank	Author	Whole-count records	Rank	Journal	Whole-count records
1	CHINA	14,695	1	HARVARD UNIVERSITY	1,087	1	SMITH GD	472	1	MEDICINE	1,170
2	UNITED STATES	3,364	2	UNIVERSITY OF BRISTOL	1,083	2	ZHANG Y	419	2	SCIENTIFIC REPORTS	653
3	UNITED KINGDOM	3,043	3	UNIVERSITY OF LONDON	962	3	WANG Y	413	3	FRONTIERS IN ENDOCRINOLOGY	467
4	GERMANY	959	4	HARVARD UNIVERSITY MEDICAL AFFILIATES	830	4	LI Y	382	4	FRONTIERS IN IMMUNOLOGY	412
5	SWEDEN	950	5	UNIVERSITY OF CAMBRIDGE	783	5	LIU Y	351	5	FRONTIERS IN GENETICS	399
6	NETHERLANDS	865	6	ZHEJIANG UNIVERSITY	684	6	BURGESS S	342	6	DISCOVER ONCOLOGY	368
7	AUSTRALIA	846	7	CENTRAL SOUTH UNIVERSITY	681	7	Li J	305	7	NATURE COMMUNICATIONS	308
8	CANADA	649	8	HARVARD MEDICAL SCHOOL	652	8	Wang J	274	8	PLOS ONE	249
9	DENMARK	613	9	IMPERIAL COLLEGE LONDON	623	9	Li X	272	9	JOURNAL OF AFFECTIVE DISORDERS	240
10	FRANCE	436	10	CAPITAL MEDICAL UNIVERSITY	622	10	Zhang L	268	10	INTERNATIONAL JOURNAL OF EPIDEMIOLOGY	230
11	ITALY	431	11	KAROLINSKA INSTITUTET	620	11	Wang L	242	11	FRONTIERS IN NUTRITION	220
12	NORWAY	412	12	SUN YAT-SEN UNIVERSITY	604	12	Chen Y	237	12	NUTRIENTS	173
13	FINLAND	389	13	SICHUAN UNIVERSITY	600	13	Chen J	234	13	FRONTIERS IN MICROBIOLOGY	167
14	SPAIN	360	14	SHANGHAI JIAO TONG UNIVERSITY	598	14	Liu J	228	14	FRONTIERS IN CARDIOVASCULAR MEDICINE	165
15	SOUTH KOREA	286	15	UNIVERSITY OF OXFORD	577	15	Zhang J	228	15	BMC MEDICINE	162

Countries/regions, institutions, and authors were counted using whole counting. Under this approach, a publication involving multiple countries/regions, institutions, or authors contributed one count to each participating unit. Therefore, the values represent whole-count participation records rather than mutually exclusive fractional publication shares. Journal counts represent the number of A-corpus records published in each journal.

At the institutional level, Harvard University (1,087 whole-count records), the University of Bristol (1,083), and the University of London (962) had the highest participation frequencies, followed by Harvard University Medical Affiliates (830) and the University of Cambridge (783) (Table 3). At the author level, Smith GD (472 whole-count records), Zhang Y (419), Wang Y (413), Li Y (382), and Liu Y (351) were the most frequently represented authors. Medicine, Scientific Reports, and Frontiers in Endocrinology contained the largest numbers of A-corpus records (Table 3).

### 3.3 Knowledge base and thematic evolution

Keyword clustering identified interrelated clusters labeled cardiovascular disease, association, genome wide association, Mendelian randomization, blood pressure, and body mass index (Figure 7). The clusters included methodological topics, cardiovascular and metabolic applications, and genomic-data-related themes.

**FIGURE 7:**
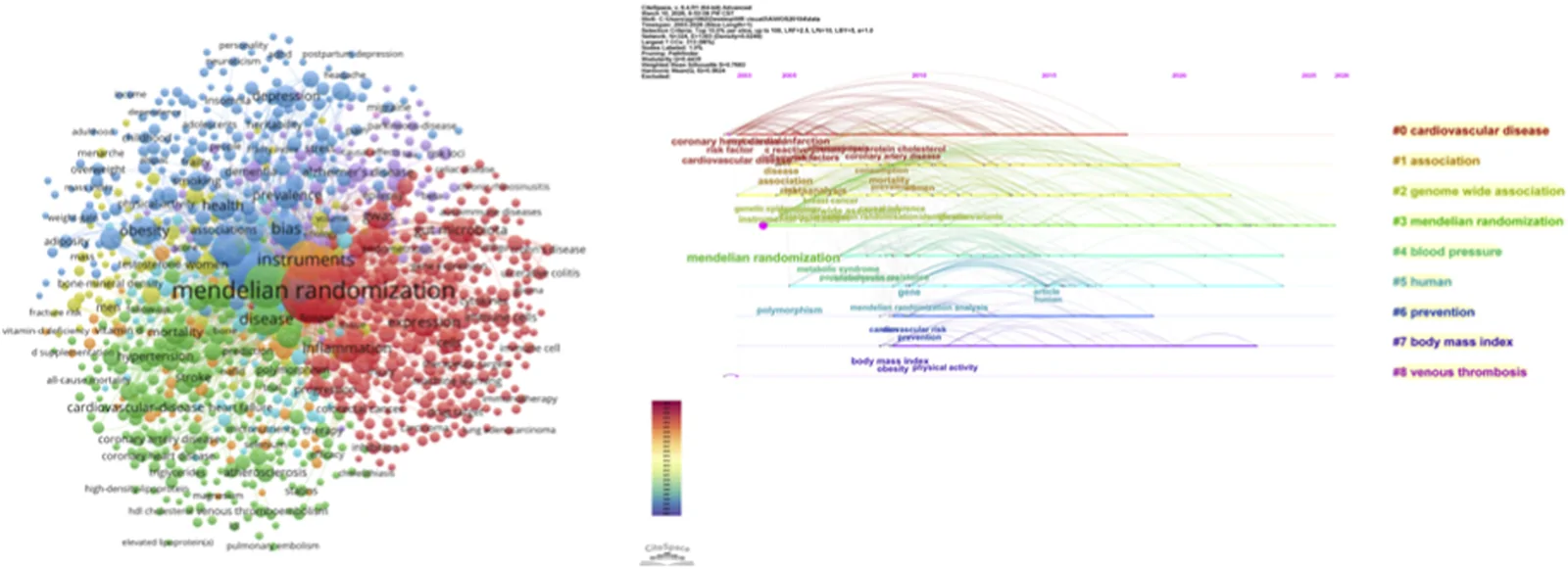
Keyword clustering and thematic evolution in the A-corpus. The visualization summarizes co-occurring keyword clusters and their distribution across the observation period; cluster labels denote the dominant terms within each group.

Timeline and burst analyses showed changes in prominent terms across the observation period (Figures 7, 8). Early burst keywords included coronary heart disease, cardiovascular disease, myocardial infarction, and instrumental variables, whereas more recent bursts included United Kingdom Biobank, pleiotropy, causal effect, ulcerative colitis, and anxiety.

**FIGURE 8:**
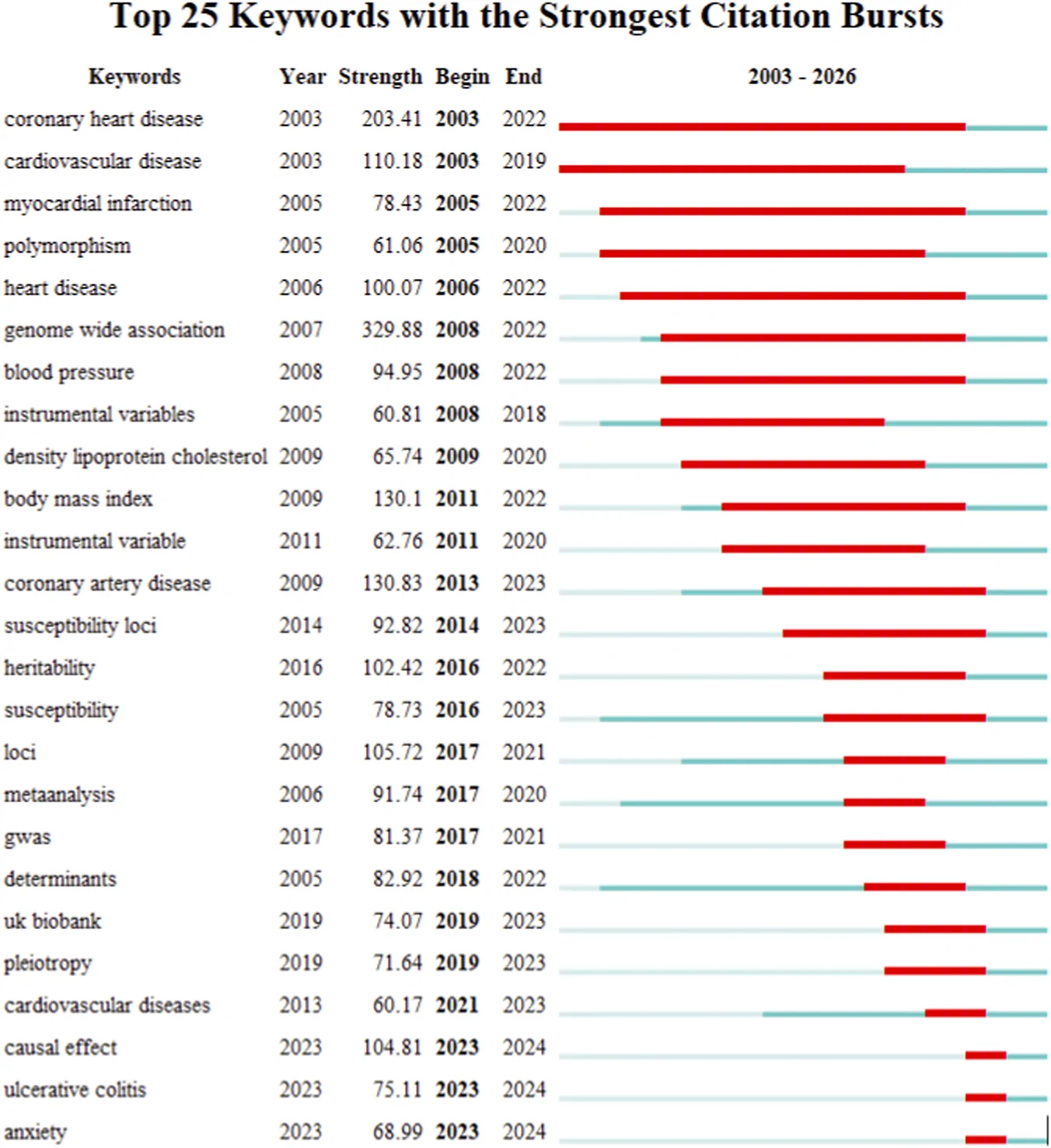
Keyword burst analysis in the A-corpus. The visualization identifies terms with marked increases in citation-period occurrence and displays the corresponding burst intervals across the observation period.

Reference co-citation analysis was regenerated after cited-reference alias merging and verification of unresolved reference-string variants. The revised network no longer contained duplicate or Bowden J variants, and the previously visible aggregate node was not retained in the labeled core network. The most prominent labeled core references included Verbanck et al. (2018), Bowden et al. (2016), Burgess et al. (2013), Lawlor et al. (2008), Bowden et al. (2015), Hemani et al. (2018), and Palmer et al. (2012) (Figure 9).

**FIGURE 9:**
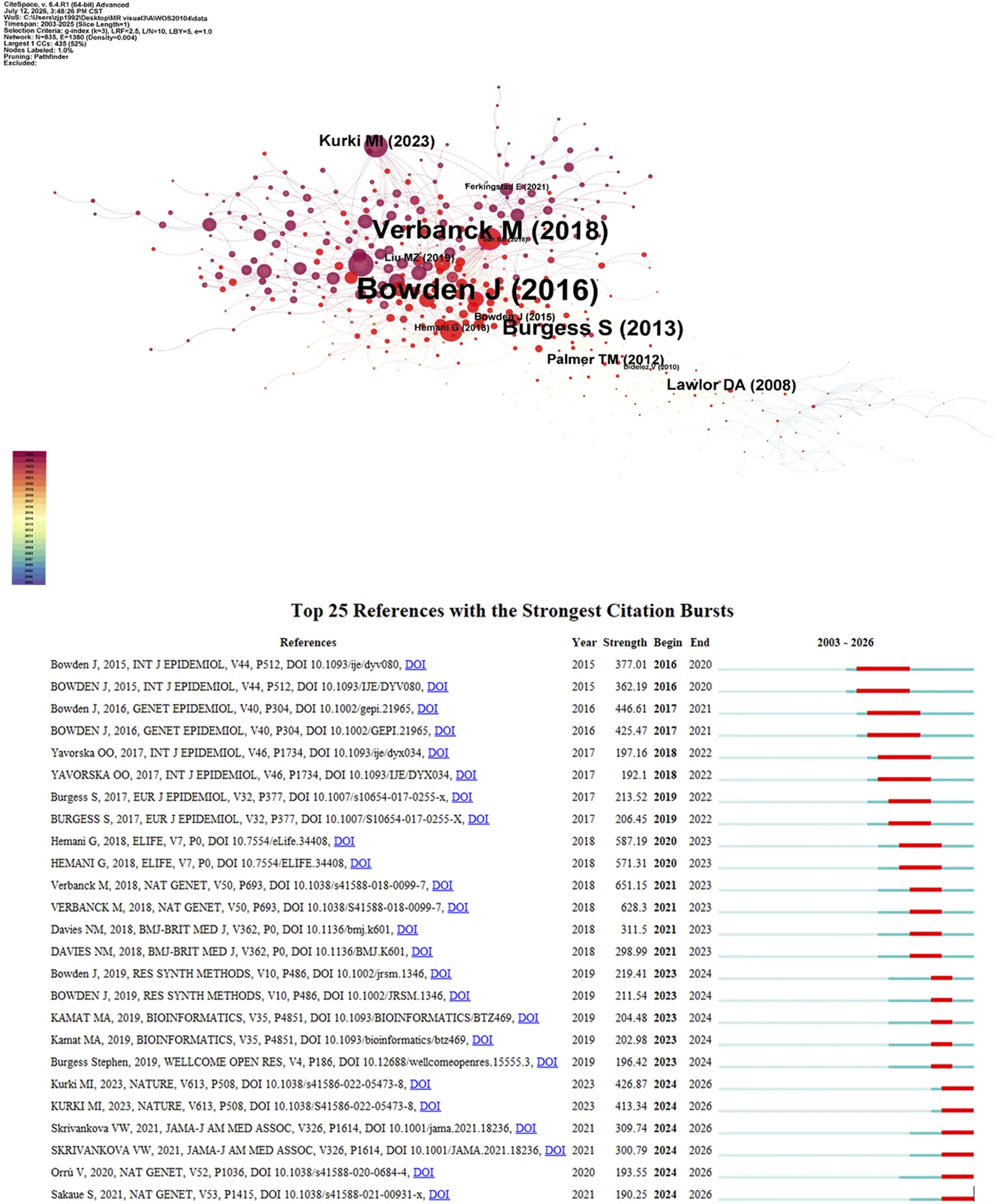
Reference co-citation structure after cited-reference alias merging. Labeled nodes identify prominent co-cited references, and connections represent co-citation relationships within the A-corpus.

### 3.4 Methodological challenges in the B-corpus

After strict dictionary-based screening, eligibility review, independent double coding, and consensus adjudication, 307 problem-driven B-corpus publications were identified (Table 4). Consensus coding results showed that the most prominent methodological challenge in contemporary MR research was horizontal pleiotropy (90 publications, 29.3%), followed by general methodological framework (48, 15.6%), molecular attribution/colocalization (41, 13.4%), reverse causation/directionality (32, 10.4%), and reporting/reproducibility/transparency (31, 10.1%) (Figure 2; Table 4). Weak instruments (26, 8.5%) also remained a highly visible concern.

**TABLE 4:** Frequency summary of the B-corpus coding results.

Category	Frequency	Percentage (%)
Panel A. Methodological challenges		
Horizontal pleiotropy	90	29.3
General methodological framework	48	15.6
Molecular attribution/colocalization	41	13.4
Reverse causation/directionality	32	10.4
Reporting/reproducibility/transparency	31	10.1
Weak instruments	26	8.5
Family-structure-related bias	14	4.6
Multivariable confounding decomposition	10	3.3
Population stratification/ancestry	7	2.3
Nonlinear/lifecourse/time-varying effects	5	1.6
Sample overlap/winner’s curse	3	1.0
Panel B. Solution pathways		
Mixed sensitivity framework	107	34.9
Reporting guideline standards	45	14.7
Omics integration framework	33	10.7
Bidirectional/directionality framework	28	9.1
Within-family or family design	19	6.2
Triangulation/external validation	15	4.9
MVMR	14	4.6
Mediation MR	14	4.6
Nonlinear MR	11	3.6
cis-MR/drug-target MR	9	2.9
Colocalization	7	2.3
MR-Egger	5	1.6
Panel C. Future priorities		
None explicitly stated	94	30.6
Reproducible reporting pipeline	45	14.7
Mechanism-aware inference	36	11.7
Omics integration	34	11.1
Clinical translation/public health application	21	6.8
Family-based designs	19	6.2
Drug-target translation	17	5.5
Triangulation with external evidence	15	4.9
Multi-ancestry validation	13	4.2
Nonlinear lifecourse MR	13	4.2
Panel D. Solution orientations		
Validity-first	138	45.0
Mechanism-aware	78	25.4
Hybrid	42	13.7
Generalizability-enhanced	32	10.4
Translation-ready	17	5.5

Each B-corpus publication contributes one mutually exclusive primary code per dimension. Percentages are based on n = 307. “None explicitly stated” means that no future-priority statement meeting the coding criteria was explicit in the analyzed text; it does not imply absence of future research needs.

The three most frequent primary challenge codes were horizontal pleiotropy, general methodological framework, and molecular attribution/colocalization.

### 3.5 Solution pathways in the B-corpus

Mixed sensitivity framework was the dominant solution pathway, accounting for 107 publications (34.9%), followed by reporting guideline standards (45, 14.7%), omics integration framework (33, 10.7%), and bidirectional/directionality framework (28, 9.1%) (Table 4). Within-family or family design (19, 6.2%), triangulation/external validation (15, 4.9%), MVMR (14, 4.6%), and mediation MR (14, 4.6%) were also represented.

The challenge-solution heatmap showed the observed cross-tabulation patterns between primary challenge and solution codes (Figure 3). Horizontal pleiotropy was most frequently paired with mixed sensitivity framework, reporting guideline standards, and MR-Egger; molecular attribution/colocalization was most frequently paired with omics integration framework, cis-MR/drug-target MR, and colocalization; and family-structure-related bias was paired predominantly with within-family or family design.

### 3.6 Future priorities and solution-oriented framework

The most frequent future-priority code was “none explicitly stated” (94 publications, 30.6%) (Table 4). This code means only that no future-priority statement meeting the coding criteria was explicit in the analyzed text; it does not indicate that the publication had no unresolved methodological issue or that no future research need existed. Among publications with an explicitly codable future direction, reproducible reporting pipeline (45, 14.7%), mechanism-aware inference (36, 11.7%), and omics integration (34, 11.1%) were the most frequent, followed by clinical translation/public health application (21, 6.8%) and family-based designs (19, 6.2%). Drug-target translation, triangulation with external evidence, and multi-ancestry validation were also represented (Table 4; Figure 4A).

Within the solution-orientation dimension, validity-first was the most frequent primary code, with 138 publications (45.0%), followed by mechanism-aware (78, 25.4%), hybrid (42, 13.7%), generalizability-enhanced (32, 10.4%), and translation-ready (17, 5.5%) (Table 4). Figure 4B presents these five descriptive orientations. The hybrid code represents publications in which two or more orientations were comparably emphasized and no single dominant orientation could be assigned.

Table 5 summarizes the coded relationships among methodological challenges, dominant solutions, remaining gaps, explicitly stated future priorities, and solution orientations. Horizontal pleiotropy was most often paired with mixed sensitivity frameworks; molecular attribution/colocalization was most often paired with multi-omics approaches; and family-structure-related bias was paired with within-family or family-design strategies. These entries summarize patterns within the coded literature and do not rank the scientific validity of the methods represented.

**TABLE 5:** Synthesis matrix of challenges, solutions, gaps, and future priorities.

Methodological challenge	No. of articles	Dominant solutions	Remaining gaps	Future priorities	Dominant orientation
Horizontal pleiotropy	90	Mixed sensitivity framework (n = 59); reporting guideline standards (n = 8); MR-egger (n = 5)	Residual horizontal pleiotropy remains difficult to rule out when robust estimators disagree or when biological pathways are poorly characterized	None explicitly stated (n = 43); mechanism-aware inference (n = 12); reproducible reporting pipeline (n = 12)	Validity-first (n = 65); hybrid (n = 8)
General methodological framework	48	Mixed sensitivity framework (n = 17); triangulation/external validation (n = 9); omics integration framework (n = 5)	Many framework papers do not translate into harmonized analytic pipelines, leaving inconsistency in routine practice and reporting	None explicitly stated (n = 15); triangulation with external evidence (n = 9); mechanism-aware inference (n = 5)	Validity-first (n = 20); hybrid (n = 15)
Molecular attribution/colocalization	41	Omics integration framework (n = 24); cis-MR/drug-target MR (n = 5); colocalization (n = 4)	Colocalization evidence is still uneven across loci and omics layers, limiting confident molecular attribution of MR signals	Omics integration (n = 22); drug-target translation (n = 7); mechanism-aware inference (n = 5)	Mechanism-aware (n = 27); translation-ready (n = 7)
Reverse causation/directionality	32	Bidirectional/directionality framework (n = 24); mixed sensitivity framework (n = 4); triangulation/external validation (n = 2)	Directionality checks remain sensitive to measurement error and correlated traits, especially in bi-directional settings	None explicitly stated (n = 9); mechanism-aware inference (n = 6); reproducible reporting pipeline (n = 5)	Mechanism-aware (n = 22); hybrid (n = 5)
Reporting/reproducibility/transparency	31	Reporting guideline standards (n = 29); mediation MR (n = 2)	Reporting standards are improving, but analytic transparency and reproducible pipelines are still inconsistently implemented across studies	Reproducible reporting pipeline (n = 24); mechanism-aware inference (n = 3); clinical/public health application (n = 3)	Validity-first (n = 25); hybrid (n = 3)
Weak instruments	26	Mixed sensitivity framework (n = 21); nonlinear MR (n = 2); cis-MR/drug-target MR (n = 1)	Instrument strength remains heterogeneous across exposures, with persistent risks of weak-instrument bias in smaller or less heritable traits	None explicitly stated (n = 20); nonlinear/lifecourse MR (n = 2); clinical/public health application (n = 1)	Validity-first (n = 21); hybrid (n = 3)
Family-structure-related bias	14	Within-family or family design (n = 14)	Within-family and related designs are still underused, limiting control of dynastic effects, assortative mating, and shared familial structure	Family-based designs (n = 14)	Generalizability-enhanced (n = 14)
Multivariable confounding decomposition	10	MVMR (n = 9); mediation MR (n = 1)	Interpretation of direct effects in multivariable MR remains challenging when exposures are highly correlated or biologically overlapping	None explicitly stated (n = 5); omics integration (n = 2); mechanism-aware inference (n = 2)	Mechanism-aware (n = 9); translation-ready (n = 1)
Population stratification/ancestry	7	Mixed sensitivity framework (n = 3); within-family or family design (n = 2); cis-MR/drug-target MR (n = 1)	Evidence remains concentrated in European-ancestry datasets, constraining cross-population validity and transferability	Multi-ancestry validation (n = 2); family-based designs (n = 2); clinical/public health application (n = 1)	Generalizability-enhanced (n = 4); hybrid (n = 1)
Nonlinear/lifecourse/time-varying effects	5	Nonlinear MR (n = 4); mediation MR (n = 1)	Nonlinear and lifecourse frameworks are still methodologically sparse and difficult to implement consistently across exposures	Nonlinear/lifecourse MR (n = 4); mechanism-aware inference (n = 1)	Mechanism-aware (n = 5)
Sample overlap/winner’s curse	3	Mixed sensitivity framework (n = 2); omics integration framework (n = 1)	Residual bias from sample overlap and winner’s curse remains insufficiently quantified in many applied two-sample MR studies	Omics integration (n = 1); none explicitly stated (n = 1); clinical/public health application (n = 1)	Mechanism-aware (n = 1); validity-first (n = 1)

The matrix summarizes dominant coded patterns and the authors’ synthesis of remaining gaps. It does not compare the scientific validity of individual methods. “None explicitly stated” denotes absence of an explicit codable future-priority statement, not absence of future research needs.

## 4 Discussion

Taken together, the A-corpus and B-corpus analyses show rapid growth in the published MR literature alongside recurrent attention to methodological validity, particularly horizontal pleiotropy. The coded literature also included solution pathways and stated priorities related to molecular attribution, omics integration, reporting, generalizability, and translation. These patterns are consistent with increasing attention to these topics within the publications analyzed, but they do not by themselves establish a field-wide developmental transition or the validity of individual MR findings.

The publication trend and collaboration networks document broad use of MR across the eligible literature. This expansion coincided with the accumulation of GWAS summary statistics, open two-sample MR resources, and the use of MR in drug-target, multi-omic, and applied clinical contexts (Davey Smith and Hemani, 2014; Hemani et al., 2018; Gill et al., 2021; Cupi et al., 2024; Schmidt et al., 2020). The large whole-count participation volume from China should be interpreted as participation frequency, not a fractional contribution share or direct evidence of methodological leadership. Likewise, network centrality describes connectedness within the analyzed metadata and does not establish scientific influence or superiority.

Within the selected B-corpus, horizontal pleiotropy was the most frequent primary methodological-challenge code. This prominence is methodologically plausible because pleiotropy bears directly on the exclusion-restriction assumption: when a genetic variant affects the outcome through pathways other than the exposure of interest, the causal interpretation of a conventional MR estimate becomes vulnerable. The frequency observed here should be interpreted as the representation of this topic in the problem-driven, methods-oriented corpus, not as an estimate of the prevalence of pleiotropic bias across all MR studies. The corresponding prominence of mixed sensitivity frameworks is consistent with methodological literature describing MR-Egger, weighted median, mode-based estimation, MR-PRESSO, multi-variant summary-data methods, and structured sensitivity analyses for assessing robustness under different assumptions (Bowden et al., 2015; Bowden et al., 2016; Hartwig et al., 2017; Verbanck et al., 2018; Burgess et al., 2017; Burgess et al., 2013).

Mixed sensitivity framework was the most frequent primary solution-pathway code in the B-corpus. Under the coding manual, this category denotes a panel of complementary estimators, diagnostics, and robustness checks rather than reliance on a single method. Its frequency indicates that such combinations were commonly foregrounded in the methods-oriented publications selected for content coding. It does not establish that this workflow is sufficient, universally used, or scientifically superior. Many of the included tools diagnose violations or assess robustness after instrument selection rather than eliminating upstream design conditions that generate bias; this distinction helps contextualize the separate attention given to preventive design, transparent reporting, and reproducible workflows.

Within the coded methodological literature, the observed future-priority patterns are consistent with increasing attention to mechanistic interpretation, generalizability, and translational application alongside conventional validity-focused work. Mechanism-aware inference and omics integration were frequent explicit priorities, while family-based and cross-context designs addressed concerns about dynastic effects, assortative mating, population stratification, and generalizability (Zhu et al., 2016; Giambartolomei et al., 2014; Brumpton et al., 2020; Skrivankova et al., 2021a; Cupi et al., 2024; Schmidt et al., 2020; Davies et al., 2019; Richmond and Davey Smith, 2022).

The four named solution orientations-validity-first, mechanism-aware, generalizability-enhanced, and translation-ready-together with the hybrid code provide a descriptive framework for organizing the dominant emphasis of publications in the B-corpus. Validity-first work focuses on bias and robustness; mechanism-aware work on biological interpretation and molecular attribution; generalizability-enhanced work on ancestry, family structure, and external validity; and translation-ready work on drug-target, clinical, or public-health applications. The framework summarizes coded emphases and should not be interpreted as a ranking of methods or evidence that any orientation is scientifically superior.

This study has several strengths. Rather than stopping at conventional bibliometric description of frequent keywords, collaboration networks, and co-citation structures, we extended the macro-level knowledge-mapping analysis by constructing a problem-driven B-corpus and prespecified coding framework. This enabled a structured description of the methodological challenges, solution pathways, and explicitly stated future priorities represented in the coded corpus while retaining a clear distinction between publication patterns and the validity of individual MR findings.

Several limitations should be considered. First, the analysis was restricted to WOSCC and Scopus and to English-language articles and reviews; publications indexed only elsewhere or published in other languages may have been missed. Second, the exact phrase-based search prioritized specificity but may have reduced comprehensiveness by excluding early or related instrumental-variable genetic studies that did not use “Mendelian randomization” or “Mendelian randomisation.” Third, dictionary-based B-corpus screening similarly prioritized methodological specificity and may have missed publications using uncommon terminology or expressing methodological issues only implicitly. Fourth, the one-primary-code-per-dimension rule facilitated mutually interpretable frequency distributions and cross-tabulations but simplified multidimensional publications and may have underrepresented secondary challenges, solutions, or priorities. Fifth, inter-coder agreement was moderate for methodological challenges and solution pathways, indicating that several categories were not sharply separable at the publication level. Although disagreements were resolved through consensus adjudication using the source text and prespecified definitions, the kappa values indicate residual subjectivity in the taxonomy and should be considered when interpreting category-level frequencies. Finally, bibliometric indicators and content coding characterize publication patterns and reported methodological emphases; they cannot determine the comparative validity of specific MR methods or the causal or clinical credibility of individual findings.

In summary, this study integrates macro-level bibliometric mapping with problem-driven content coding to characterize publication patterns, recurrent methodological concerns, reported solution pathways, and explicitly stated future priorities in MR research. Horizontal pleiotropy and related validity concerns remained prominent, while reproducible reporting, mechanism-aware inference, omics integration, generalizability, and translation were represented among the coded solutions and priorities. These findings describe emphases in the published literature; they do not establish which MR methods are scientifically superior or whether individual MR findings are causally or clinically valid.

## References

[B1] Aria M. Cuccurullo C. (2017). Bibliometrix: an R-tool for comprehensive science mapping analysis. J. Informetr. 11, 959–975. 10.1016/j.joi.2017.08.007

[B2] Bowden J. Davey Smith G. Burgess S. (2015). Mendelian randomization with invalid instruments: effect estimation and bias detection through egger regression. Int. J. Epidemiol. 44, 512–525. 10.1093/ije/dyv080 26050253 PMC4469799

[B3] Bowden J. Davey Smith G. Haycock P. C. Burgess S. (2016). Consistent estimation in Mendelian randomization with some invalid instruments using a weighted median estimator. Genet. Epidemiol. 40, 304–314. 10.1002/gepi.21965 27061298 PMC4849733

[B4] Brumpton B. Sanderson E. Hartwig F. P. Harrison S. Vie G. A. Cho Y. (2020). Avoiding dynastic, assortative mating, and population stratification biases in Mendelian randomization through within-family analyses. Nat. Commun. 11, 3519. 10.1038/s41467-020-17117-4 32665587 PMC7360778

[B5] Burgess S. Thompson S. G. (2011). CRP CHD genetics collaboration. Avoiding bias from weak instruments in Mendelian randomization studies. Int. J. Epidemiol. 40, 755–764. 10.1093/ije/dyr036 21414999

[B6] Burgess S. Butterworth A. Thompson S. G. (2013). Mendelian randomization analysis with multiple genetic variants using summarized data. Genet. Epidemiol. 37, 658–665. 10.1002/gepi.21758 24114802 PMC4377079

[B7] Burgess S. Timpson N. J. Ebrahim S. Davey Smith G. (2015). Mendelian randomization: where are we now and where are we going? Int. J. Epidemiol. 44, 379–388. 10.1093/ije/dyv108 26085674

[B8] Burgess S. Davies N. M. Thompson S. G. (2016). Bias due to participant overlap in two-sample Mendelian randomization. Genet. Epidemiol. 40, 597–608. 10.1002/gepi.21998 27625185 PMC5082560

[B9] Burgess S. Bowden J. Fall T. Ingelsson E. Thompson S. G. (2017). Sensitivity analyses for robust causal inference from mendelian randomization analyses with multiple genetic variants. Epidemiology 28, 30–42. 10.1097/EDE.0000000000000559 27749700 PMC5133381

[B10] Burgess S. Davey Smith G. Davies N. M. Dudbridge F. Gill D. Glymour M. M. (2023). Guidelines for performing Mendelian randomization investigations: update for summer 2023. Wellcome Open Res. 4, 186. 10.12688/wellcomeopenres.15555.3 32760811 PMC7384151

[B11] Carter A. R. Sanderson E. Hammerton G. Richmond R. C. Davey Smith G. Heron J. (2021). Mendelian randomisation for mediation analysis: current methods and challenges for implementation. Eur. J. Epidemiol. 36, 465–478. 10.1007/s10654-021-00757-1 33961203 PMC8159796

[B12] Chen C. (2006). CiteSpace II: detecting and visualizing emerging trends and transient patterns in scientific literature. J. Am. Soc. Inf. Sci. Technol. 57, 359–377. 10.1002/asi.20317

[B13] Cupido A. J. Zhou M. Lusis A. J. Seldin M. M. (2024). Specific approaches and limitations in (Multi)-omic Mendelian randomization. J. Lipid Res. 65, 100619. 10.1016/j.jlr.2024.100619 39147365 PMC11467659

[B14] Davey Smith G. Ebrahim S. (2003). Mendelian randomization': can genetic epidemiology contribute to understanding environmental determinants of disease? Int. J. Epidemiol. 32, 1–22. 10.1093/ije/dyg070 12689998

[B15] Davey Smith G. Hemani G. (2014). Mendelian randomization: genetic anchors for causal inference in epidemiological studies. Hum. Mol. Genet. 23, R89–R98. 10.1093/hmg/ddu328 25064373 PMC4170722

[B16] Davies N. M. Holmes M. V. Davey Smith G. (2018). Reading Mendelian randomisation studies: a guide, glossary, and checklist for clinicians. BMJ 362, k601. 10.1136/bmj.k601 30002074 PMC6041728

[B17] Davies N. M. Howe L. J. Brumpton B. Havdahl A. Evans D. M. Davey Smith G. (2019). Within family Mendelian randomization studies. Hum. Mol. Genet. 28, R170–R179. 10.1093/hmg/ddz204 31647093

[B18] Giambartolomei C. Vukcevic D. Schadt E. E. Franke L. Hingorani A. D. Wallace C. (2014). Bayesian test for colocalisation between pairs of genetic association studies using summary statistics. PLoS Genet. 10, e1004383. 10.1371/journal.pgen.1004383 24830394 PMC4022491

[B19] Gill D. Georgakis M. K. Walker V. M. Schmidt A. F. Gkatzionis A. Freitag D. F. (2021). Mendelian randomization for studying the effects of perturbing drug targets. Wellcome Open Res. 6, 16. 10.12688/wellcomeopenres.16544.2 33644404 PMC7903200

[B20] Hartwig F. P. Davey Smith G. Bowden J. (2017). Robust inference in summary data Mendelian randomization via the zero modal pleiotropy assumption. Int. J. Epidemiol. 46, 1985–1998. 10.1093/ije/dyx102 29040600 PMC5837715

[B21] Haycock P. C. Burgess S. Wade K. H. Bowden J. Relton C. Davey Smith G. (2016). Best (but oft-forgotten) practices: the design, analysis, and interpretation of Mendelian randomization studies. Am. J. Clin. Nutr. 103, 965–978. 10.3945/ajcn.115.118216 26961927 PMC4807699

[B22] Hemani G. Tilling K. Davey Smith G. (2017). Orienting the causal relationship between imprecisely measured traits using GWAS summary data. PLoS Genet. 13, e1007081. 10.1371/journal.pgen.1007081 29149188 PMC5711033

[B23] Hemani G. Zheng J. Elsworth B. Wade K. H. Haberland V. Baird D. (2018). The MR-Base platform supports systematic causal inference across the human phenome. eLife 7, e34408. 10.7554/eLife.34408 29846171 PMC5976434

[B24] Jiang T. Gill D. Butterworth A. S. Burgess S. (2023). An empirical investigation into the impact of winner's curse on Mendelian randomization estimates. Int. J. Epidemiol. 52, 1209–1220. 10.1093/ije/dyac233 PMC10396423 36573802

[B25] Katan M. B. (1986). Apolipoprotein E isoforms, serum cholesterol, and cancer. Lancet 1, 507–508. 10.1016/S0140-6736(86)92972-7 2869248

[B26] Lawlor D. A. Harbord R. M. Sterne J. A. Timpson N. Davey Smith G. (2008). Mendelian randomization: using genes as instruments for making causal inferences in epidemiology. Stat. Med. 27, 1133–1163. 10.1002/sim.3034 17886233

[B27] Morrison J. Knoblauch N. Marcus J. H. Stephens M. He X. (2020). Mendelian randomization accounting for correlated and uncorrelated pleiotropic effects using genome-wide summary statistics. Nat. Genet. 52, 740–747. 10.1038/s41588-020-0631-4 32451458 PMC7343608

[B28] Page M. J. McKenzie J. E. Bossuyt P. M. Boutron I. Hoffmann T. C. Mulrow C. D. (2021). The PRISMA 2020 statement: an updated guideline for reporting systematic reviews. BMJ 372, n71. 10.1136/bmj.n71 33782057 PMC8005924

[B41] Palmer T. M. Lawlor D. A. Harbord R. M. Sheehan N. A. Tobias J. H. Timpson N. J. (2012). Using multiple genetic variants as instrumental variables for modifiable risk factors. Stat. Methods Med. Res. 21, 223–242. 10.1177/0962280210394459 21216802 PMC3917707

[B29] Richmond R. C. Davey Smith G. (2022). Mendelian randomization: concepts and scope. Cold Spring Harb. Perspect. Med. 12, a040501. 10.1101/cshperspect.a040501 34426474 PMC8725623

[B30] Saccaro L. F. Gasparini S. Rutigliano G. (2022). Applications of Mendelian randomization in psychiatry: a comprehensive systematic review. Psychiatr. Genet. 32, 199–213. 10.1097/YPG.0000000000000327 36354137 PMC9648985

[B31] Sanderson E. Davey Smith G. Windmeijer F. Bowden J. (2019). An examination of multivariable Mendelian randomization in the single-sample and two-sample summary data settings. Int. J. Epidemiol. 48, 713–727. 10.1093/ije/dyy262 30535378 PMC6734942

[B32] Schmidt A. F. Finan C. Gordillo-Maranon M. Asselbergs F. W. Freitag D. F. Patel R. S. (2020). Genetic drug target validation using Mendelian randomisation. Nat. Commun. 11, 3255. 10.1038/s41467-020-16969-0 32591531 PMC7320010

[B33] Skrivankova V. W. Richmond R. C. Woolf B. A. R. Davies N. M. Swanson S. A. VanderWeele T. J. (2021a). The STROBE-MR statement: strengthening the reporting of observational studies in epidemiology using Mendelian randomization. JAMA 326, 1614–1621. 10.1001/jama.2021.18236 34698778

[B34] Skrivankova V. W. Richmond R. C. Woolf B. A. R. Davies N. M. Swanson S. A. VanderWeele T. J. (2021b). Strengthening the reporting of observational studies in epidemiology using Mendelian randomisation (STROBE-MR): explanation and elaboration. BMJ 375, n2233. 10.1136/bmj.n2233 34702754 PMC8546498

[B35] Staley J. R. Burgess S. (2017). Semiparametric methods for estimation of a nonlinear exposure-outcome relationship using instrumental variables with application to Mendelian randomization. Genet. Epidemiol. 41, 341–352. 10.1002/gepi.22041 28317167 PMC5400068

[B36] van Eck N. J. Waltman L. (2010). Software survey: VOSviewer, a computer program for bibliometric mapping. Scientometrics 84, 523–538. 10.1007/s11192-009-0146-3 20585380 PMC2883932

[B37] Verbanck M. Chen C. Y. Neale B. Do R. (2018). Detection of widespread horizontal pleiotropy in causal relationships inferred from Mendelian randomization between complex traits and diseases. Nat. Genet. 50, 693–698. 10.1038/s41588-018-0099-7 29686387 PMC6083837

[B38] Wallace C. (2021). A more accurate method for colocalisation analysis allowing for multiple causal variants. PLoS Genet. 17, e1009440. 10.1371/journal.pgen.1009440 34587156 PMC8504726

[B39] Zhong D. Cheng H. (2024). Application of Mendelian randomization in the discovery of risk factors for coronary heart disease from 2009 to 2023: a bibliometric review. Clin. Cardiol. 47, e24154. 10.1002/clc.24154 37724687 PMC10765999

[B40] Zhu Z. Zhang F. Hu H. Bakshi A. Robinson M. R. Powell J. E. (2016). Integration of summary data from GWAS and eQTL studies predicts complex trait gene targets. Nat. Genet. 48, 481–487. 10.1038/ng.3538 27019110

