# Supplementary material for: Methodological challenges, solution pathways, and future priorities in Mendelian randomization research: a bibliometric and content analysis
Source: Front Genet. 2026 Oct 26;17:1853737. doi: 10.3389/fgene.2026.1853737 (PMC13744922; doi:10.3389/fgene.2026.1853737)
Supplement: Supplementary file 1 [file DataSheet1.zip › MR_B_corpus_reproducibility_package_20260919/Supplementary_Tables_S1-S4.docx]

**Supplementary Table S1. Terminology dictionary and coding manual for B-corpus screening and four-dimensional content coding**

## Panel A. Methodological challenge codes

Primary code identifies the main methodological problem or bias source addressed by the publication.

| **Category / standard code** | **Synonyms / search terms** | **Operational definition** | **Inclusion rule** | **Exclusion / stop rule** | **Example method/source anchors** |
| --- | --- | --- | --- | --- | --- |
| **Horizontal pleiotropy** | horizontal pleiotropy; pleiotropic effect; directional pleiotropy; invalid instrument; MR-Egger intercept; MR-PRESSO; outlier pleiotropy; heterogeneity as pleiotropy signal | Primary concern that genetic instruments affect the outcome through pathways other than the exposure, violating the exclusion-restriction assumption. | Assign when the paper primarily develops, evaluates, detects, corrects, or discusses pleiotropic bias or pleiotropy-robust inference in MR. | Do not assign when pleiotropy appears only as a routine sensitivity check in an otherwise exposure-outcome application without methodological emphasis. | Bowden et al. 2015; Verbanck et al. 2018; Hartwig et al. 2017; Morrison et al. 2020. |
| **General methodological framework** | methodological framework; best practice; primer; review; guidance; protocol; analytic workflow; causal inference framework; methodological overview | Broad methodological synthesis or framework-level discussion that does not primarily focus on a single bias source or single solution method. | Assign when the main contribution is a general framework, guidance article, tutorial, checklist, or broad synthesis of MR methods and interpretation. | If the paper clearly prioritizes one named bias or method, assign that more specific category instead. | Davey Smith and Hemani 2014; Davies et al. 2018; Burgess et al. 2015; Skrivankova et al. 2021. |
| **Molecular attribution / colocalization** | colocalization; co-localisation; molecular attribution; locus attribution; shared causal variant; eQTL; pQTL; mQTL; SMR; HEIDI; transcriptome; proteome; methylation | Primary challenge concerns assigning an MR signal to a causal gene, molecular layer, locus, tissue, or biological mechanism rather than to linkage disequilibrium or nearby correlated signals. | Assign when the paper focuses on colocalization, molecular target attribution, omics-based locus interpretation, or distinguishing causal molecular signals. | Do not assign when omics data are used only as an exposure without explicit attribution, colocalization, or mechanism-identification focus. | Giambartolomei et al. 2014; Zhu et al. 2016; Cupido et al. 2024; Schmidt et al. 2020. |
| **Reverse causation / directionality** | reverse causation; bidirectional MR; directionality; Steiger filtering; causal direction; reciprocal causation; bidirectional analysis | Primary concern is whether the inferred causal direction is correct or whether the exposure-outcome direction may be reversed or reciprocal. | Assign when directionality testing, bidirectional MR, Steiger directionality, or causal-order assessment is the central methodological issue. | Do not assign when a bidirectional analysis is included only as a routine applied analysis and not discussed as a methodological issue. | Steiger directionality literature; Hemani et al. 2018; Davey Smith and Hemani 2014. |
| **Reporting / reproducibility / transparency** | reporting guideline; STROBE-MR; checklist; reproducibility; transparency; code availability; data availability; open science; workflow; quality appraisal | Primary challenge concerns incomplete reporting, unclear analytic choices, lack of data/code transparency, or limited reproducibility of MR studies. | Assign when the paper develops, applies, or evaluates reporting standards, reproducible workflows, transparent pipelines, or quality-assessment practices. | Do not assign when a paper merely provides data availability or code availability statements without making reporting/reproducibility a substantive focus. | Skrivankova et al. 2021; PRISMA-style flow reporting; STROBE-MR guidance. |
| **Weak instruments** | weak instrument; F statistic; instrument strength; weak-instrument bias; variance explained; relevance assumption; instrument selection threshold | Primary concern is insufficient instrument-exposure association strength, which may bias MR estimates or reduce inferential precision. | Assign when the paper focuses on weak-instrument bias, instrument-strength assessment, instrument selection, or related finite-sample problems. | Do not assign when F statistics are only reported descriptively in an applied MR paper without methodological emphasis. | Burgess and Thompson 2011; Lawlor et al. 2008; Davies et al. 2018. |
| **Family-structure-related bias** | within-family MR; sibling design; family-based design; dynastic effect; assortative mating; population stratification within family; parental genotype; genetic nurture | Primary challenge concerns bias from family structure, dynastic effects, assortative mating, or shared familial environment that conventional population MR may not control. | Assign when within-family, sibling, trio, parental-genotype, or family-based MR designs are central to the article. | Do not assign solely for ancestry adjustment or principal components without family-structure design or discussion. | Brumpton et al. 2020; within-family MR literature. |
| **Multivariable confounding decomposition** | multivariable MR; MVMR; direct effect; correlated exposures; mediation; confounding decomposition; pleiotropy through measured traits | Primary challenge concerns separating direct effects or decomposing correlated exposures/pathways when multiple candidate exposures are biologically or statistically related. | Assign when the main issue is interpretation of direct effects, correlated exposures, or decomposition using MVMR or related methods. | If mediation is explicitly the main method-oriented topic, assign the solution pathway as mediation MR but keep challenge code here only if decomposition is the challenge focus. | Sanderson et al. 2019; Carter et al. 2021; Burgess et al. MVMR literature. |
| **Population stratification / ancestry** | population stratification; ancestry; trans-ethnic; cross-ancestry; multi-ancestry; genetic ancestry; population structure; transferability | Primary challenge concerns ancestry-related confounding, cross-population validity, or limited transferability of MR evidence across populations. | Assign when ancestry, stratification, transferability, cross-ancestry validation, or non-European generalizability is the central methodological issue. | Do not assign when ancestry is only a sample descriptor and not part of the methodological argument. | Davey Smith and Hemani 2014; within-family and multi-ancestry MR discussions. |
| **Nonlinear / lifecourse / time-varying effects** | nonlinear MR; non-linear MR; lifecourse; time-varying exposure; developmental timing; threshold effect; age-specific effect | Primary challenge concerns heterogeneity of causal effects across exposure levels, life stages, or time-varying trajectories. | Assign when nonlinear estimation, lifecourse MR, time-varying exposure interpretation, or threshold effects are the main methodological problem. | Do not assign when age/sex subgroup analyses are routine and not framed as nonlinear or lifecourse MR methodology. | Staley and Burgess 2017; lifecourse MR methodological literature. |
| **Sample overlap / winner's curse** | sample overlap; overlapping samples; winner's curse; discovery GWAS; overfitting; bias in two-sample MR; non-independent samples | Primary challenge concerns bias arising from overlap between exposure and outcome samples or inflated discovery-stage associations used as instruments. | Assign when sample overlap, winner's curse, discovery/replication separation, or two-sample dependency is the central methodological issue. | Do not assign when sample overlap is only briefly acknowledged as a limitation without methodological development or evaluation. | Burgess et al. two-sample MR bias literature; instrument-discovery and winner's-curse discussions. |

## Panel B. Solution pathway codes

Primary code identifies the principal methodological strategy or response pathway emphasized by the publication.

| **Category / standard code** | **Synonyms / search terms** | **Operational definition** | **Inclusion rule** | **Exclusion / stop rule** | **Example method/source anchors** |
| --- | --- | --- | --- | --- | --- |
| **Mixed sensitivity framework** | sensitivity analysis; robustness checks; multi-estimator framework; IVW; MR-Egger; weighted median; weighted mode; MR-PRESSO; heterogeneity; leave-one-out | Use of a panel of complementary estimators, diagnostics, and robustness checks to evaluate the stability of MR estimates under different assumptions. | Assign when the paper emphasizes a combined sensitivity/robustness workflow rather than a single named method as the principal solution. | If one named method is clearly the central methodological contribution, assign that specific method category instead. | Bowden et al. 2015; Bowden et al. 2016; Verbanck et al. 2018; Hartwig et al. 2017. |
| **Reporting guideline standards** | STROBE-MR; reporting checklist; guideline; transparency standard; code/data reporting; reproducible report; methodological quality | Standardized reporting, checklist-based, or reproducibility-oriented solution pathway for improving transparency and evaluation of MR evidence. | Assign when the main solution is a reporting guideline, checklist, or standardized transparent workflow. | Do not assign when a paper merely mentions a guideline without evaluating, applying, or advocating it as a solution. | Skrivankova et al. 2021; STROBE-MR; reproducible reporting guidance. |
| **Omics integration framework** | multi-omics; eQTL; pQTL; mQTL; transcriptomics; proteomics; methylomics; SMR; omics MR; molecular QTL | Integration of one or more omics layers with MR to support mechanism identification, molecular prioritization, or biological interpretation. | Assign when omics integration is the primary solution pathway for interpreting, validating, or prioritizing MR signals. | Do not assign when a single omics trait is analyzed as a routine exposure without an integrative or mechanistic framework. | Zhu et al. 2016; Cupido et al. 2024; Schmidt et al. 2020. |
| **Bidirectional / directionality framework** | bidirectional MR; reverse MR; Steiger directionality; causal direction; reciprocal causation; directionality testing | Analytic solution for assessing causal direction, including forward and reverse MR or directionality filtering. | Assign when directionality testing is the main methodological solution. | Do not assign when bidirectional MR is a minor applied addition with no methods-oriented interpretation. | Steiger directionality framework; Hemani et al. 2018. |
| **Within-family or family design** | within-family MR; sibling MR; family-based MR; trio design; parental genotype; genetic nurture; dynastic effects | Design-based solution using family structures to reduce confounding from dynastic effects, assortative mating, population stratification, or shared environment. | Assign when family-based design is the main solution pathway. | Do not assign to ordinary ancestry adjustment or unrelated-sample analyses. | Brumpton et al. 2020. |
| **Triangulation / external validation** | triangulation; external validation; replication; negative control; alternative data source; convergent evidence; multiple methods | Use of independent evidence streams, alternative designs, or external datasets to corroborate or contextualize MR findings. | Assign when external validation or triangulation is framed as the principal solution for improving credibility. | Do not assign when replication is only reported as routine sensitivity analysis without methodological emphasis. | Triangulation frameworks; Davey Smith and Hemani 2014; MR best-practice guidance. |
| **MVMR** | multivariable MR; MVMR; direct effect; multiple exposures; correlated risk factors; conditional instrument strength | MR framework estimating direct effects of one exposure conditional on other exposures, often used for correlated traits or pathway decomposition. | Assign when multivariable MR is the central solution method. | Do not assign if MVMR is only one component of a larger mixed sensitivity panel and not the dominant focus. | Sanderson et al. 2019. |
| **Mediation MR** | mediation MR; two-step MR; indirect effect; mediator; pathway effect; causal mediation | MR-based solution for estimating or evaluating indirect pathways through mediators. | Assign when mediation or indirect-effect estimation is the primary solution pathway. | Do not assign to general MVMR unless explicit mediation/indirect effect is emphasized. | Carter et al. 2021. |
| **Nonlinear MR** | nonlinear MR; non-linear MR; fractional polynomial; spline; threshold; exposure strata; nonlinear exposure-outcome relationship | Analytic solution for estimating non-linear or threshold causal effects of exposures on outcomes. | Assign when nonlinear causal estimation is the principal methodological solution. | Do not assign to routine subgroup analyses unless explicitly framed as nonlinear MR. | Staley and Burgess 2017. |
| **cis-MR / drug-target MR** | cis-MR; cis-region MR; drug-target MR; pharmacological target; genetic drug target validation; protein target; pQTL target | Use of variants in or near genes encoding drug targets, or target-associated molecular instruments, to inform drug-target validation and translation. | Assign when cis-region or drug-target MR is the main solution pathway. | Do not assign to broad multi-omics work unless the principal goal is drug-target validation. | Gill et al. 2021; Schmidt et al. 2020. |
| **Colocalization** | colocalization; coloc; co-localisation; shared causal variant; locus colocalization; Bayesian colocalization | Statistical assessment of whether two association signals at a locus are likely to share a causal variant, used to strengthen molecular attribution. | Assign when colocalization is the principal method or solution pathway. | Do not assign when colocalization is only briefly mentioned under broader omics integration. | Giambartolomei et al. 2014. |
| **MR-Egger** | MR-Egger; Egger regression; intercept test; InSIDE; directional pleiotropy | Regression-based robust MR estimator and diagnostic for directional pleiotropy under the InSIDE assumption. | Assign when MR-Egger itself is the central method being developed, evaluated, or emphasized. | Do not assign when MR-Egger is only one estimator within a broader mixed sensitivity framework. | Bowden et al. 2015. |

## Panel C. Future-priority codes

Primary code identifies the main forward-looking methodological or translational priority explicitly articulated by the publication.

| **Category / standard code** | **Synonyms / search terms** | **Operational definition** | **Inclusion rule** | **Exclusion / stop rule** | **Example method/source anchors** |
| --- | --- | --- | --- | --- | --- |
| **None explicitly stated** | no future direction; no explicit agenda; no forward-looking priority; no recommendation | The article does not articulate a clear future methodological priority beyond its immediate analysis or conclusion. | Assign when future needs are absent, generic, or too vague to map to another future-priority category. | Do not assign if the paper explicitly calls for a recognizable priority such as multi-ancestry validation, reporting pipelines, or mechanism-aware inference. | Routine method papers or reviews without explicit forward-looking agenda. |
| **Reproducible reporting pipeline** | reproducible pipeline; reporting guideline; STROBE-MR; code sharing; data sharing; workflow transparency; open science | Future priority centered on standardized, transparent, and reproducible MR design, analysis, reporting, and data/code sharing. | Assign when the future agenda emphasizes reporting, reproducibility, protocolization, transparency, or workflow standardization. | Do not assign to papers that merely comply with reporting standards but do not identify reproducibility as a future priority. | Skrivankova et al. 2021; MR reporting guidance. |
| **Mechanism-aware inference** | mechanism-aware; biological mechanism; pathway; mediation; molecular mechanism; causal pathway; interpretation | Future priority focusing on moving from causal direction to mechanistic explanation and pathway interpretation. | Assign when the paper emphasizes understanding biological pathways, mediators, mechanisms, or causal chains as future work. | Do not assign to generic mentions of biology without explicit methodological or mechanistic priority. | Carter et al. 2021; colocalization and omics-MR literature. |
| **Omics integration** | multi-omics; transcriptomics; proteomics; methylomics; metabolomics; eQTL; pQTL; mQTL; systems genetics | Future priority calling for integration of multiple omics layers to improve mechanism discovery, target prioritization, or biological interpretation. | Assign when future work explicitly emphasizes multi-omics or QTL integration. | Do not assign when omics are merely used in the current study without being framed as a future priority. | Zhu et al. 2016; Cupido et al. 2024. |
| **Clinical translation / public health application** | clinical translation; public health; implementation; intervention; guideline translation; prevention; policy; decision support | Future priority aimed at translating MR evidence into clinical interpretation, prevention, public-health decision-making, or policy. | Assign when translational application, clinical utility, or public-health relevance is the explicit forward-looking focus. | Do not assign when clinical disease context is only the topic of exposure-outcome analysis without translational agenda. | Gill et al. 2021; drug-target and public-health MR discussions. |
| **Family-based designs** | within-family; family-based MR; sibling design; parental genotype; dynastic effect; assortative mating; genetic nurture | Future priority emphasizing wider use or development of family-based designs to improve causal validity and generalizability. | Assign when the paper calls for within-family, sibling, or family-based MR as future work. | Do not assign if family-based design is the current method but not a future priority. | Brumpton et al. 2020. |
| **Drug-target translation** | drug target; target validation; pharmacological perturbation; therapeutic target; target prioritization; cis-pQTL; genetic proxy for drug effect | Future priority focused on using MR to validate, prioritize, or interpret therapeutic targets and drug-development decisions. | Assign when drug-target validation or therapeutic translation is the explicit future direction. | Do not assign to general clinical translation unless drug-target development is central. | Gill et al. 2021; Schmidt et al. 2020. |
| **Triangulation with external evidence** | triangulation; external evidence; replication; causal triangulation; negative control; randomized trial comparison; observational validation | Future priority emphasizing validation of MR evidence using independent designs, datasets, or evidence streams. | Assign when the paper explicitly calls for triangulation, replication across designs, or external validation as future work. | Do not assign to routine replication unless framed as a priority for methodological credibility. | Triangulation and best-practice MR literature. |
| **Multi-ancestry validation** | multi-ancestry; cross-ancestry; trans-ethnic; non-European; diversity; ancestry validation; population transferability | Future priority focused on improving evidence across ancestries and populations to enhance transferability and reduce Eurocentric bias. | Assign when future work emphasizes cross-ancestry replication, non-European datasets, or population diversity. | Do not assign when ancestry is only a sample descriptor or adjustment covariate. | MR generalizability and ancestry-diversity literature. |
| **Nonlinear / lifecourse MR** | nonlinear MR; lifecourse MR; time-varying MR; threshold; exposure timing; developmental window | Future priority focused on advancing methods or applications that consider nonlinearity, timing, or life-stage-specific causal effects. | Assign when the future agenda explicitly calls for nonlinear, lifecourse, or time-varying MR. | Do not assign to current nonlinear analysis unless future development is explicitly articulated. | Staley and Burgess 2017; lifecourse MR discussions. |

## Panel D. Solution-orientation codes

Primary code abstracts the publication into a high-level orientation. The hybrid orientation is a single mutually exclusive category, not a multi-label relaxation.

| **Category / standard code** | **Synonyms / search terms** | **Operational definition** | **Inclusion rule** | **Exclusion / stop rule** | **Example method/source anchors** |
| --- | --- | --- | --- | --- | --- |
| **Validity-first** | validity; bias control; pleiotropy; weak instruments; robustness; sensitivity analysis; reporting validity; causal credibility | Dominant orientation is protecting causal inference validity by detecting, reducing, or transparently reporting sources of MR bias. | Assign when the primary emphasis is robust estimation, bias detection, sensitivity analysis, reporting quality, or validity of causal claims. | If the dominant emphasis is mechanism, generalizability, translation, or balanced multi-orientation without a dominant focus, assign the more appropriate orientation or hybrid. | Pleiotropy-robust estimators; weak-instrument assessment; STROBE-MR reporting. |
| **Mechanism-aware** | mechanism; pathway; mediation; omics; colocalization; biological interpretation; molecular attribution | Dominant orientation is explaining biological mechanisms or pathways behind causal effects, including omics and molecular attribution. | Assign when the paper primarily aims to understand pathways, mediators, causal biology, or molecular mechanisms. | Do not assign to purely validity-focused work that uses mechanism language only as background. | Colocalization; SMR; mediation MR; multi-omics integration. |
| **Generalizability-enhanced** | generalizability; multi-ancestry; external validity; family design; transferability; diversity; population structure | Dominant orientation is improving transferability, external validity, or robustness of evidence across populations, ancestries, or family structures. | Assign when ancestry diversity, family-based design, population transferability, or external validity is the primary emphasis. | Do not assign when generalizability is mentioned only as a limitation without methodological focus. | Within-family MR; multi-ancestry validation; cross-population MR. |
| **Translation-ready** | translation; drug target; therapeutic target; clinical implementation; public health; intervention; policy | Dominant orientation is moving MR evidence toward therapeutic, clinical, public-health, or policy decision-making. | Assign when the paper primarily aims to support drug-target validation, clinical translation, intervention prioritization, or public-health application. | Do not assign to disease-context applications without explicit translational orientation. | Drug-target MR; genetic target validation; clinical/public-health translation. |
| **Hybrid** | hybrid; bridging orientation; integrated framework; validity + mechanism; mechanism + translation; generalizability + validity | An independent mutually exclusive primary orientation code assigned when two or more pure orientations are comparably emphasized and no single dominant orientation can be identified. | Assign only when the article substantively bridges two or more pure orientations and coders cannot justify a single dominant orientation after reviewing the title, abstract, keywords, key evidence, and full text if needed. | Do not assign merely because the article mentions multiple orientations. If one orientation is clearly dominant, code that pure orientation instead. | Integrated methods papers bridging robustness, mechanistic attribution, generalizability, and/or translation. |

## Decision hierarchy and adjudication notes

1. Screen first for explicit methodological, solution-oriented, reporting/reproducibility, or future-priority focus. Routine exposure-outcome MR applications were not retained unless they substantively addressed a methodological issue.

2. Apply the one-primary-code rule independently within each dimension. Multiple secondary ideas may be noted, but only the primary code is used for frequency and cross-tabulation analyses.

3. Prefer specific codes over broad framework codes when a publication clearly focuses on a named bias or named method.

4. Assign "none explicitly stated" only within the future-priority dimension when no explicit forward-looking agenda is present.

5. Assign "hybrid" only within the solution-orientation dimension and only when two or more pure orientations are comparably emphasized and no single dominant orientation can be identified.

6. During consensus adjudication, disagreements were resolved by rechecking the title, abstract, keywords, key-evidence excerpt, full text where necessary, and the definitions in this coding manual.

**Abbreviations:** CAUSE, Causal Analysis Using Summary Effect estimates; eQTL, expression quantitative trait locus; HEIDI, heterogeneity in dependent instruments; IVW, inverse-variance weighted; MR, Mendelian randomization; MR-PRESSO, MR Pleiotropy RESidual Sum and Outlier; MVMR, multivariable Mendelian randomization; pQTL, protein quantitative trait locus; SMR, summary-data-based Mendelian randomization; STROBE-MR, Strengthening the Reporting of Observational Studies in Epidemiology Using Mendelian Randomization.

**Supplementary Material**

**Supplementary Table S2. Screening and exclusion log for construction of the B-corpus**

*Panel A. A-corpus to B-corpus screening flow*

| **Screening stage** | **Records, n** | **Notes** |
| --- | --- | --- |
| Records exported from Web of Science Core Collection | 20,104 | Full bibliographic records exported in plain-text format. |
| Records exported from Scopus | 19,715 | BibTeX records exported from Scopus. |
| Total records before deduplication | 39,819 | WOSCC + Scopus records before merging and deduplication. |
| Duplicates removed by DOI matching | 18,376 | Exact DOI-based duplicate removal after DOI normalization. |
| Duplicates removed by exact title + publication year | 97 | Residual duplicates identified by standardized title-year matching. |
| Duplicates removed by title + first author + publication year | 0 | No additional duplicates removed at this step. |
| Final deduplicated A-corpus | 21,346 | Main bibliometric corpus. |
| Records screened using the strict terminology dictionary | 21,346 | Dictionary applied to titles, abstracts, and keywords. |
| Records without strict dictionary hits | 10,748 | Not carried forward to B-corpus eligibility review. |
| Records with >=1 strict dictionary hit | 10,598 | Candidate records for B-corpus eligibility review. |
| Candidate records excluded after eligibility review | 10,291 | Excluded with primary reason categories in Panel B. |
| Final B-corpus included for content coding | 307 | Final problem-driven content-analysis corpus. |

Note: The B-corpus was derived from the A-corpus through strict dictionary screening followed by eligibility review. Percentages may not total 100% owing to rounding.

*Panel B. Primary exclusion reasons among dictionary-hit records not retained in the B-corpus*

| **Primary exclusion reason** | **Excluded records, n** | **% of excluded records** | **% of dictionary-hit records** |
| --- | --- | --- | --- |
| Routine exposure-outcome MR application | 6,437 | 62.5 | 60.7 |
| Omics/drug-target MR application without methodological evaluation | 3,326 | 32.3 | 31.4 |
| Method-related signal present but insufficient primary B-corpus focus | 412 | 4.0 | 3.9 |
| GWAS/resource study with MR only as secondary or peripheral component | 68 | 0.7 | 0.6 |
| No explicit MR methodological challenge/solution/future-priority focus | 44 | 0.4 | 0.4 |
| Non-eligible publication type or protocol/commentary | 3 | 0.0 | 0.0 |
| Disease-/topic-specific review, not MR-methodological | 1 | 0.0 | 0.0 |
| Total excluded after eligibility review | 10,291 | 100.0 | 97.1 |
| Final B-corpus retained | 307 | - | 2.9 |

Note: Exclusion reasons were assigned hierarchically. When more than one exclusion reason was plausible, the primary reason was assigned according to the dominant reason for non-inclusion in the B-corpus. The category 'routine exposure-outcome MR application' refers to studies in which MR was applied to a specific exposure-outcome association and standard sensitivity analyses were reported, but the article did not primarily develop, evaluate, compare, or synthesize MR methodological challenges or solution pathways.

**Supplementary Table S3. Inter-coder reliability of B-corpus coding**

| **Coding dimension** | **Records double-coded, n** | **Percent agreement, %** | **Cohen's kappa** | **Interpretation** |
| --- | --- | --- | --- | --- |
| Methodological challenge | 307 | 50.5 | 0.434 | Moderate |
| Solution pathway | 307 | 63.5 | 0.531 | Moderate |
| Future priority | 307 | 73.6 | 0.703 | Substantial |
| Solution orientation | 307 | 92.8 | 0.897 | Almost perfect |

Note. All 307 B-corpus records were independently coded by two reviewers before consensus adjudication. Cohen's kappa was calculated using unweighted nominal categories. Percent agreement is reported as an additional descriptive measure. Final analyses were based on consensus codes after disagreement resolution.

**Supplementary Table S4. Software parameters for bibliometric and visualization analyses**

**Table note:** The parameters below are based on the VOSviewer screenshots for organization, country, and keyword networks and the CiteSpace screenshots for keyword clustering/burst detection and reference co-citation analysis. Full/whole counting was recorded for VOSviewer maps because the maps were created with the standard full-counting workflow unless otherwise specified in the create-map step.

| **Analysis / figure** | **Software** | **Data source** | **Analysis type / node type** | **Counting / weighting method** | **Threshold / selection criterion** | **Normalization, layout, clustering, and pruning** | **Network metrics / output** |
| --- | --- | --- | --- | --- | --- | --- | --- |
| Figure 2. Annual publication trend | R statistical software | Deduplicated A-corpus | Publication year | Not applicable | All deduplicated records included | Annual aggregation by publication year; graphical presentation in R | Annual publication counts summarized by year |
| Figure 3A. Country collaboration network | VOSviewer 1.6.20 | A-corpus source files imported into VOSviewer | Co-authorship; unit of analysis: countries/regions | Full counting / whole counting; node weight = documents | Minimum documents of a country = 5; minimum citations of a country = 0; 120 of 187 countries met the threshold | Association-strength normalization; default layout; attraction = 2; repulsion = 1; clustering resolution = 1.00; minimum cluster size = 1; merge small clusters = yes; maximum label length = 30; maximum displayed lines = 1000 | Items = 120; clusters = 16; links = 2,603; total link strength = 82,914 |
| Figure 3B. Institutional collaboration network | VOSviewer 1.6.20 | A-corpus source files imported into VOSviewer | Co-authorship; unit of analysis: organizations/institutions | Full counting / whole counting; node weight = documents | Minimum documents of an organization = 60; minimum citations of an organization = 0; 275 of 9,526 organizations met the threshold | Association-strength normalization; default layout; attraction = 2; repulsion = 1; clustering resolution = 1.00; minimum cluster size = 1; merge small clusters = yes; maximum label length = 30; maximum displayed lines = 1000 | Items displayed = 259; clusters = 6; links = 9,537; total link strength = 71,241 |
| Figure 4A. Keyword co-occurrence network | VOSviewer 1.6.20 | A-corpus source files imported into VOSviewer | Co-occurrence; unit of analysis: keywords | Full counting / whole counting; node weight = occurrences | Minimum occurrences of a keyword = 60; 289 of 16,289 keywords met the threshold | Association-strength normalization; default layout; attraction = 2; repulsion = 0; clustering resolution = 1.00; minimum cluster size = 1; merge small clusters = yes; maximum label length = 30; maximum displayed lines = 1000 | Items = 289; clusters = 10; links = 14,055; total link strength = 92,628 |
| Figure 4B. Keyword clustering and thematic timeline | CiteSpace 6.4.R1 Advanced | WOSCC records within the A-corpus; WOS20104 data folder | Node type: keywords; time slicing = 2003–2025; slice length = 1 year | Not applicable | Selection criteria: top 10.0% per slice, up to 100; LRF = 2.5; L/N = 10; LBY = 5; e = 1.0 | Pruning = Pathfinder; nodes labeled = 1.0%; cluster/time-zone visualization generated in CiteSpace | Network N = 324; E = 1,303; density = 0.0249; largest connected component = 313 nodes (96%); modularity Q = 0.4435; weighted mean silhouette S = 0.7683; harmonic mean (Q, S) = 0.5624 |
| Figure 5. Top emerging research fronts / keyword burst detection | CiteSpace 6.4.R1 Advanced | WOSCC records within the A-corpus; WOS20104 data folder | Node type: keywords; time slicing = 2003–2025; slice length = 1 year | Not applicable | Same keyword-network settings as Figure 4B: top 10.0% per slice, up to 100; LRF = 2.5; L/N = 10; LBY = 5; e = 1.0; top 25 strongest bursts reported | Pruning = Pathfinder; CiteSpace burst-detection output used to report burst strength, begin year, and end year | Keyword network metrics: N = 324; E = 1,303; density = 0.0249; largest connected component = 313 nodes (96%); modularity Q = 0.4435; weighted mean silhouette S = 0.7683 |
| Figure 6. Reference co-citation structure and intellectual evolution after cited-reference alias merging | CiteSpace 6.4.R1 Advanced | WOSCC cited references; WOS20104 data folder | Node type: cited reference; time slicing = 2003–2025; slice length = 1 year | Not applicable | Selection criteria: g-index (k = 3); LRF = 2.5; L/N = 10; LBY = 5; e = 1.0 | Pruning = Pathfinder; nodes labeled = 1.0%; cited-reference variants manually inspected and merged using CiteSpace alias function before rerun | Network N = 835; E = 1,380; density = 0.004; largest connected component = 435 nodes (52%) |
| Figure 7. Distribution of methodological challenges | R 4.5.2 software | Final consensus B-corpus | Primary methodological-challenge code | Consensus primary code | All 307 B-corpus records included | Frequency bar chart in R | Frequencies and percentages by challenge category |
| Figure 8. Challenge–solution heatmap | R 4.5.2 software | Final consensus B-corpus | Methodological challenge × solution pathway | Consensus primary code | All 307 B-corpus records included | Heatmap based on cross-tabulated cell counts | Challenge–solution cross-tabulated counts |
| Figure 9A. Future-priority temporal trends | R 4.5.2 software | Final consensus B-corpus | Future priority × publication year | Consensus primary code | All 307 B-corpus records included | Line plot by publication year in R | Annual counts by future-priority category |
| Figure 9B. Five-orientation solution-oriented framework | R 4.5.2 software | Final consensus B-corpus | Solution orientation | Consensus primary code | All 307 B-corpus records included | Conceptual framework based on consensus-coded category frequency | Validity-first, n = 138; mechanism-aware, n = 78; hybrid, n = 42; generalizability-enhanced, n = 32; translation-ready, n = 17 |
